# Single-cell analysis reveals context-dependent, cell-level selection of mtDNA

**DOI:** 10.1038/s41586-024-07332-0

**Published:** 2024-04-24

**Authors:** Anna V. Kotrys, Timothy J. Durham, Xiaoyan A. Guo, Venkata R. Vantaku, Sareh Parangi, Vamsi K. Mootha

**Affiliations:** 1 Howard Hughes Medical Institute and Department of Molecular Biology, Massachusetts General Hospital, Harvard Medical School, Boston, MA USA; 2 Broad Institute of MIT and Harvard, Cambridge, MA USA; 3 Department of Surgery, Massachusetts General Hospital, Harvard Medical School, Boston, MA USA; 4 Cancer Center, Massachusetts General Hospital, Boston, MA USA

**Keywords:** Genomics, Sequencing

## Abstract

Heteroplasmy occurs when wild-type and mutant mitochondrial DNA (mtDNA) molecules co-exist in single cells^1^. Heteroplasmy levels change dynamically in development, disease and ageing^2,3^, but it is unclear whether these shifts are caused by selection or drift, and whether they occur at the level of cells or intracellularly. Here we investigate heteroplasmy dynamics in dividing cells by combining precise mtDNA base editing (DdCBE)^4^ with a new method, SCI-LITE (single-cell combinatorial indexing leveraged to interrogate targeted expression), which tracks single-cell heteroplasmy with ultra-high throughput. We engineered cells to have synonymous or nonsynonymous complex I mtDNA mutations and found that cell populations in standard culture conditions purge nonsynonymous mtDNA variants, whereas synonymous variants are maintained. This suggests that selection dominates over simple drift in shaping population heteroplasmy. We simultaneously tracked single-cell mtDNA heteroplasmy and ancestry, and found that, although the population heteroplasmy shifts, the heteroplasmy of individual cell lineages remains stable, arguing that selection acts at the level of cell fitness in dividing cells. Using these insights, we show that we can force cells to accumulate high levels of truncating complex I mtDNA heteroplasmy by placing them in environments where loss of biochemical complex I activity has been reported to benefit cell fitness. We conclude that in dividing cells, a given nonsynonymous mtDNA heteroplasmy can be harmful, neutral or even beneficial to cell fitness, but that the ‘sign’ of the effect is wholly dependent on the environment.

## Abstract (web summary)

A new method for tracking single-cell heteroplasmy, called SCI-LITE, is combined with mitochondrial DNA base editing to reveal principles of heteroplasmy dynamics in dividing cells.

## Main

Mitochondria contain a high copy, maternally transmitted genome that encodes key components of the oxidative phosphorylation (OXPHOS) machinery. The mtDNA has a higher mutation rate than the nuclear genome^5^, and heteroplasmy occurs when mutant mtDNA molecules co-exist with wild type^1^. Inherited mtDNA mutations can cause devastating OXPHOS diseases^6^, whereas somatic mtDNA mutations are observed in many cancers, and in a small subset of rare tumours may even drive tumorigenesis^7–9^. Moreover, mutant mtDNA heteroplasmy has been reported to accumulate with ageing^2^ and proposed to contribute to degenerative diseases^2,10^.

Heteroplasmy dynamics are incredibly complex in cell culture and in vivo^1^. In maternally transmitted diseases, levels of heteroplasmy can vary across tissues within the same individual and can be dramatically different between siblings^11^. In cell culture, mutant mtDNA load can be decreased via ‘heteroplasmic shifting’. Previous studies have utilized bulk populations of cybrid cells to show that using fuels that force dependence on mitochondrial OXPHOS can select against deleterious mtDNA alleles^12–14^. Mechanisms underlying these heteroplasmy dynamics remain unclear. In particular, it is uncertain whether heteroplasmic shifting is driven by drift or selection and, if the latter, whether it is operating at the level of cell fitness or intracellularly (mtDNA turnover and mitophagy).

Historically, most studies of heteroplasmy utilized bulk populations of cells carrying naturally occurring mtDNA mutations, because tools such as CRISPR do not work on mtDNA^15^. Ideally, one would be able to carefully investigate heteroplasmy dynamics of synonymous variants alongside those that are nonsynonymous. Moreover, being able to investigate heteroplasmy at the level of single cells is important, as 50% bulk heteroplasmy may arise from two extreme scenarios: either (1) each cell in the population is approximately 50% heteroplasmic, or (2) one-half of the cells contain 100% wild-type mtDNA, whereas the other half contains 100% mutant mtDNA. Yet, most studies reported to date have analysed bulk populations of cells, as single-cell heteroplasmy analysis remains so cumbersome.

Here we combine a recently introduced mtDNA base-editing technology^4^ with SCI-LITE, a highly scalable, sensitive, cost-effective and flexible tool for measuring specific transcripts in single cells. We engineered and monitored the heteroplasmy dynamics of synonymous and nonsynonymous variants at the single-cell level over time, both across populations of cells and within clonal lineages. Our analyses demonstrate a key role for environment-dependent selection that acts at the level of cell fitness to shape heteroplasmy dynamics in populations of dividing cells.

## Single-cell mtDNA analysis via SCI-LITE

Multiple ‘well-based’ and ‘droplet-based’ single-cell approaches have been described, yet none of them is well suited for large-scale single-cell mtDNA heteroplasmy analysis. Well-based approaches require high manual labour, whereas droplet-based approaches are prohibitively expensive and require specialized microfluidic devices. An alternative family of single-cell genomics approaches leverages single-cell combinatorial indexing (‘SCI’)^16–18^ to generate combinatorial molecular tags during split–pool barcoding rounds that probabilistically identify individual cells. SCI approaches do not require encapsulation of cells into droplets, but instead treat each cell as an independent compartment.

SCI-LITE adapts the combinatorial indexing approach of sci-RNA sequencing (sci-RNA-seq) and Split-seq^17,18^ to capture selected transcripts, increasing scalability and reducing sequencing costs. Here we applied SCI-LITE to interrogate mtDNA heteroplasmy, which can be read out at the RNA level^19,20^, and we multiplexed SCI-LITE to interrogate both mtDNA and nuclear transcripts in one experiment.

**Fig. 1:**
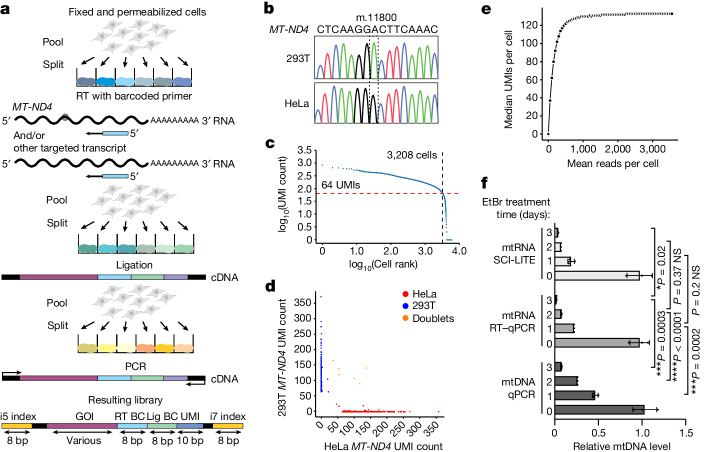
SCI-LITE enables ultra-high-throughput analysis of targeted transcripts in single cells. **a**, Fixed and permeabilized cells are distributed into wells in which targeted transcripts are labelled with well-specific barcodes. The first barcode is added during reverse transcription (RT). The second barcode and the UMI are added in the ligation step after the first round of pooling and splitting. The third and fourth barcodes are added by PCR after the second round of pooling and splitting. Lengths are presented in base pairs (bp). BC, barcode; GOI, gene of interest; Lig, ligation. Schematics of cells in part **a** were created using BioRender (https://biorender.com). **b**–**e**, Barnyard experiment. Reads were assigned to the HeLa or 293T cell line based on the unique sequence of the *MT-ND4* transcript (**b**). Knee plot showing UMI per cell count indicating the number of barcodes corresponding to single cells (**c**). Barnyard plot showing the number of HeLa and 293T UMIs per cell (**d**). The two cell lines were mixed at equal ratios at the beginning of the experiment. Cells with alleles assigned to one cell line are considered singlets and coloured in blue (293T) or red (HeLa). Cells with mixed alleles are considered doublets and coloured in orange. Median UMIs detected per cell are presented as a function of raw sequencing reads (**e**). **f**, mtDNA and mtRNA depletion with EtBr measured by qPCR of mtDNA, RT–qPCR of mtRNA and SCI-LITE. *n* = 3 biological replicates. Error bars reflect the mean ± s.d. ****P ≤ 0.0001, ***P ≤ 0.001, *P ≤ 0.1 and not significant (NS) > 0.05 by Student’s unpaired two-tailed *t*-test. Source Data

**Extended Data Fig. 1:**
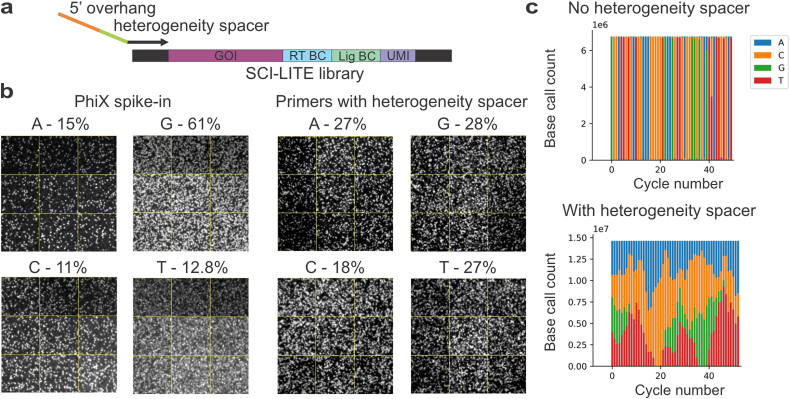
Heterogeneity primers increase nucleotide diversity during sequencing of SCI-LITE libraries. **a**, Schematic of heterogeneity primer design for amplifying SCI-LITE libraries. **b**, Representative zoomed-in cluster images from a single cycle of Illumina sequencing, for a run with PhiX spike-in (left) and for a run with a library prepared with heterogeneity spacers (right) generated with the Illumina Sequencing Analysis Viewer. The subplots are labeled with the called base and the percentage of clusters called for each base **c**, Base diversity at each bp of read 1 based on sequencing data from libraries prepared with or without heterogeneity primers.

In SCI-LITE, fixed and permeabilized cells underwent three split–pool rounds of barcoding (Fig. 1a; Methods): (1) cells were distributed into multi-well plates, and cDNA was generated in situ using target-specific barcoded reverse transcription primers; (2) cells were pooled and redistributed into new multi-well plates, and ligation was performed to add a second barcode and unique molecular identifier (UMI) to the cDNA; and (3) cells were pooled and redistributed into new multi-well plates, where they were lysed and used for nested PCR. The first round of PCR introduced partial sequencing adapters and a ‘heterogeneity spacer’ of variable lengths that allowed sequencing of otherwise very similar amplicons out of phase^21^ (Extended Data Fig. 1). Then, a second PCR was performed to introduce dual Illumina indexes, which constitute the third and fourth barcodes. After sequencing, each transcript was assigned to a particular cell based on its combination of the four barcodes (Fig. 1a). This barcoding strategy can be readily scaled with additional rounds of pool–split ligations. We developed a freely available software package for analysing SCI-LITE data (see the ‘Code availability’ section).

To evaluate the ability of SCI-LITE to generate uniquely labelled cells and to evaluate the doublet rate, we performed a stringent version of the ‘Barnyard experiment’^18,22^. We mixed two different human cell lines, HeLa and 293T, that can be distinguished by a naturally occurring homoplasmic mtDNA polymorphism in the *MT-ND4* transcript (m.11800G (HeLa) and m.11800A (293T)) (Fig. 1b), and we assessed the proportion of reads per cell aligning uniquely to each cell line. We observed good separation of reads: 94.2% of all reads matched either the HeLa or 293T allele, and after counting the number of UMIs for each barcode combination and using a ‘knee plot’ to filter for the uniquely barcoded single cells with sufficient coverage (at least 64 UMIs per cell), 99.2% of cells were unambiguously assigned to a cell line (Fig. 1c,d). During sequential split–pool rounds, it is possible for two cells to traverse the same path by random chance. Here the theory predicts a ‘doublet’ rate of approximately 2% overall, representing the sum of ‘homotypic’ and ‘heterotypic’ doublets^22^. In our data, we can detect and therefore reliably determine the heterotypic doublet rate, which is 0.8% (see Supplementary Discussion and Supplementary Fig. 1 for details about heterotypic and homotypic doublets). We performed analysis similar to other single-cell methods^18^, and we found that our libraries for the Barnyard experiment are sequenced to sufficient depth; on average, we obtained 3,620 reads per cell (median of 3,213) with an average of 148 UMIs per cell (median of 130) (Fig. 1e).

Next, we examined to what extent SCI-LITE can detect differences in mtDNA or mtRNA abundance. We treated cells with ethidium bromide (EtBr), which inhibits both mtDNA replication and transcription^23,24^, and therefore may influence mtDNA and mtRNA levels differently. We treated cells for 24, 48 and 72 h with EtBr and analysed the resulting mtDNA levels in bulk by quantitative PCR (qPCR), and the mtRNA levels both in bulk by qPCR with reverse transcription (RT–qPCR) and in single cells by SCI-LITE. As expected, EtBr treatment caused a gradual decrease of mtDNA and mtRNA abundance over time. We found that, in EtBr, bulk mtRNA levels were lower than bulk mtDNA levels, consistent with previous work showing that mtDNA and mtRNA levels are correlated but not always identical^24,25^. We found that SCI-LITE precisely reflects mtRNA levels (Fig. 1f). Collectively, these Barnyard results show that SCI-LITE enables quantitative analysis of transcript variants in single cells at scale.

## Bimodal distribution of engineered heteroplasmy

**Fig. 2:**
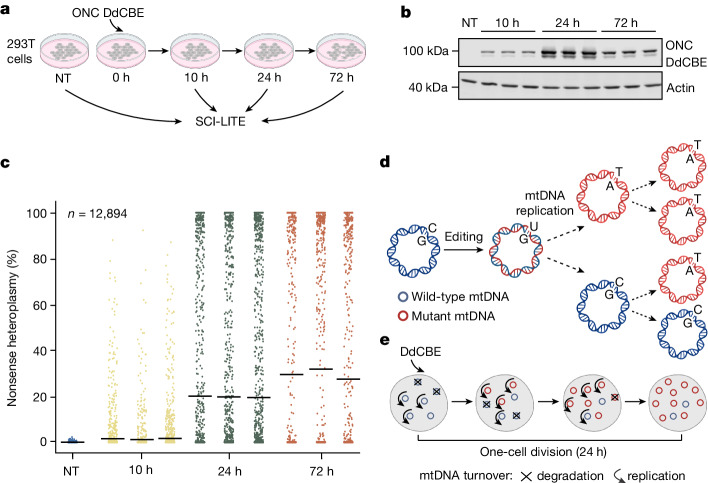
mtDNA base editing leads to a bimodal distribution of heteroplasmy. **a**, Schematic overview of the SCI-LITE experiment. 293T cells were transfected with the ONC DdCBE, introducing a nonsense mutation in the *MT-ND4* gene. Cells were cultured for 10 h, 24 h and 72 h, harvested and used for SCI-LITE. **b**, Western blot of ONC DdCBEs, showing expression of FLAG-tagged DdCBE halves (see Supplementary Fig. 2 for uncropped images). Actin was used as a loading control. *n* = 3 biological replicates are shown. **c**, Single-cell heteroplasmy in 293T cells interrogated using SCI-LITE. *n* = 3 biological replicates of edited cells are shown. Lines represent the mean for single biological replicates. Dots represent single cells. NT, not treated. **d**, Proposed model for heteroplasmy installation using mtDNA base editing. DdCBEs convert cytosine to uracil within double-stranded mtDNA molecules. Replication of one edited mtDNA molecule leads to the formation of one mutated and one wild-type mtDNA molecule. Further editing and replication of wild-type mtDNA molecules leads to formation of 50% mutant and 50% wild-type molecules. Replication of mutated molecules results in 100% mutant mtDNA molecules. **e**, For a single cell, assuming a fully active editor, after *n* rounds of mtDNA replication, we would expect that the heteroplasmy of the cell *H* = 1 − (1/2)^*n*^. Under this simple model, after just a few rounds of mtDNA replication in the face of an active base editor, a single cell will achieve a heteroplasmy level approaching 100%, but only if the mtDNA replication and turnover rate exceeds the cell division rate. Schematics in parts **a**,**d**,**e** were created using BioRender (https://biorender.com). Source Data

**Extended Data Fig. 2:**
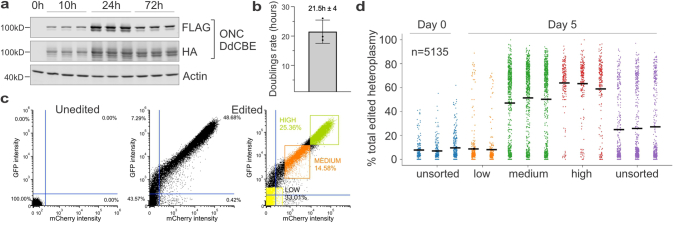
Expression of DdCBEs correlates to heteroplasmy levels. **a**, Western blots of ONC DdCBEs showing expression of FLAG-tagged right-TALE DdCBE and HA-tagged left-TALE DdCBE. Actin was used as a loading control. Data for *n* = 3 biological replicates are shown. **b**, Population doubling rate in 293 T cells transfected with DdCBEs, *n* = 3 biological replicates. Error bars reflect the mean ± s.d. **c**-**d**, 293 T cells were transfected with LHON DdCBE and sorted based on the fluorescence intensity of eGFP and mCherry reporters into low, medium and high groups. eGFP and mCherry intensity was used as a proxy for the expression of the editors **c**, Sorting strategy. Representative gating plots are shown. Graphs were generated using FCS Express software. **d**, Single-cell heteroplasmy measured by SCI-LITE, *n* = 3 biological replicates of edited cells are shown, except day 5 low where *n* = 2 biological replicates. Lines represent the mean for single biological replicates. Dots represent single cells. Source Data

In our original work which introduced DdCBE^4^, we found that installing a truncating, oncocytoma-associated *MT-ND4* mutation using the MT-ND4 DdCBE (hereafter ONC DdCBE) resulted in cell populations approaching 50% bulk heteroplasmy, and here we investigated the heteroplasmy distribution of the edited cells. We transfected 293T cells with the ONC DdCBE as we did previously^4^, but this time we performed SCI-LITE at 10, 24 and 72 h later (Fig. 2a). The editors began to be expressed at the 10-h timepoint, and their expression peaked at 24 h (Fig. 2b, Extended Data Fig. 2a and Supplementary Fig. 2). Bulk analysis revealed heteroplasmy comparable with what we previously reported^4^; however, SCI-LITE analysis revealed that DdCBE editing resulted in the generation of a striking bimodal distribution of heteroplasmy (Fig. 2c). Over the course of 72 h, or approximately three cell divisions (293T cell division rate is 21.5 ± 4 h; Extended Data Fig. 2b), we observed a gradual increase in the mean heteroplasmy level at each timepoint; however, this gradual increase was driven by individual edited cells that transitioned quickly from low to high heteroplasmy (Fig. 2c). Our finding is in line with previous reports showing very different levels of heteroplasmy in individual clones isolated from bulk populations that had been edited by mitochondrial zinc finger deaminases^26^ or transcription-activator-like effector (TALE)-linked deaminases^27^.

In our previous work, we showed that mtDNA replication is required for mtDNA editing^4^. We proposed that mutations are fixed by the action of the replicative polymerase, which installs an A opposite to the DdCBE-introduced U to resolve the U•G intermediate into the T•A base pair. Because (1) the DdCBE can edit only one strand of mtDNA, (2) mtDNA replication is necessary to fix introduced mtDNA edits, and (3) mtDNA replication is semi-conservative, we would expect a maximum of 50% editing efficiency within one cell division, assuming every mtDNA molecule is edited and then replicated once. The current SCI-LITE analysis, however, reveals that some cells can achieve extremely high levels of heteroplasmy, far exceeding 50%, within one cell division, that is, 24 h after introducing the editors (Fig. 2c). This is to be expected if there are several rounds of mtDNA replication or turnover within a single cell cycle (Fig. 2d and Extended Data Fig. 2b). Indeed, this is in line with previous findings suggesting that mtDNA replication and degradation occurs at random in mammalian cells, and that mtDNA turnover rates are high^28,29^.

Our results led to the hypothesis that the striking bimodality is simply due to the expression and activity of editors. To test this hypothesis, we turned to newly developed editors fused with the fluorescent markers eGFP and mCherry^30^. We transfected 293T cells with the MT-ND4.2 DdCBE (hereafter LHON DdCBE) to install a missense mutation in the *MT-ND4* gene associated with Leber’s hereditary optic neuropathy (LHON), then sorted cells based on their intensity of eGFP and mCherry fluorescence (Extended Data Fig. 2c) and performed SCI-LITE. Our analysis revealed that heteroplasmy level is strongly correlated with DdCBE expression level (Supplementary Fig. 2d). Given this strong correlation, we used the DdCBE expression level as a proxy for heteroplasmy; in this paper, we refer to these sorted samples as being of high or low heteroplasmy. We also confirmed the heteroplasmy levels for all samples by next-generation sequencing.

## Active selection against nonsynonymous mtDNA

**Fig. 3:**
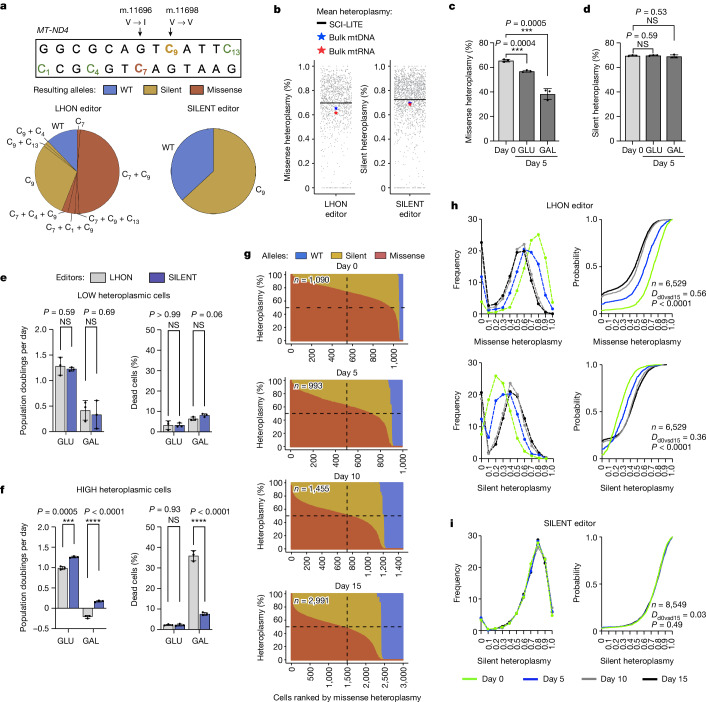
Heteroplasmic shifting operates on nonsynonymous but not silent mtDNA variants. 293T cells were transfected with LHON or SILENT DdCBEs, and sorted based on the expression of the editors reflected by the fluorescence intensity of the eGFP and mCherry reporters. **a**, Alleles and their frequency introduced by LHON and SILENT DdCBEs. Missense indicates an on-target missense G11696A edit, and Silent indicates an on-target silent C11698T edit. **b**, Single-cell heteroplasmy in edited cells interrogated using SCI-LITE or by amplicon sequencing of bulk mtDNA and bulk mtRNA. Dots represent single cells. **c**,**d**, Heteroplasmy levels assessed by next-generation sequencing of *MT-ND4* amplicon in high heteroplasmic cells treated with the LHON (**c**) or the SILENT (**d**) editor cultured in media containing glucose (GLU) or galactose (GAL). **e**,**f**, Population doublings and viability in LHON-edited and SILENT-edited low heteroplasmic (**e**) and high heteroplasmic (**f**) cells cultured in media containing either glucose or galactose. For **c**–**f**, *n* = 3 independent biological replicates. Error bars reflect the mean ± s.d. ****P ≤ 0.0001, ***P ≤ 0.001 and NS > 0.05, by Student’s unpaired two-tailed *t*-test. **g**, Joint distribution of missense and silent heteroplasmy changes over time. The graphs are sorted on the *x* axis by missense heteroplasmy and show the missense and silent heteroplasmy introduced by the LHON DdCBE in single cells measured by SCI-LITE. Each column represents one cell, with the stacked colours representing the percent missense, silent and wild-type heteroplasmy in red, yellow and blue, respectively. The dashed lines are for reference and indicate the midpoints of the *x* and *y* axes. **h**,**i**, Single-cell heteroplasmy in LHON-edited (**h**) and SILENT-edited (**i**) cells. Cells were grown for 5, 10 and 15 days and were subjected to SCI-LITE. The graphs on the left show the binned relative frequency and the graphs on the right show cumulative distributions of *MT-ND4* missense and silent heteroplasmy for *n* = 3 independent biological replicates. The Kolmogorov–Smirnov test was used to calculate *D* statistics and *P* values. Source Data

We next sought to investigate heteroplasmy dynamics of nonsynonymous versus synonymous mtDNA variants, with the goal of directly testing whether selection or drift operates against these variants. To this end, we treated cells with one of two editors: either our LHON DdCBE that introduces a missense mutation (m.11696G > A Val313Ile) in the *MT-ND4* gene encoding a subunit of complex I, or a SILENT DdCBE that introduces a synonymous mutation 2 bp away (m.11698C > T Val313Val). Of note, the LHON DdCBE not only introduces the on-target missense mutation but also introduces the same synonymous *MT-ND4* mutation as the SILENT DdCBE as an off-target (in *trans*) or a bystander (in *cis*) product (Fig. 3a). Hence, individual cells treated with the LHON DdCBE adventitiously contain a mixture of mtDNA molecules with missense, silent or both missense and silent mutations, allowing us to directly examine their single-cell dynamics with SCI-LITE. We found that the mean heteroplasmy levels measured by SCI-LITE matched the mean heteroplasmy values measured in bulk mtDNA and mtRNA following application of these editors (Fig. 3b).

To determine whether selection or drift operates against nonsynonymous and synonymous mtDNA variants, we cultured LHON-edited and SILENT-edited cells in media containing either glucose or galactose as the sole sugar source. Cells with mitochondrial OXPHOS dysfunction exhibit a cell fitness defect when cultured in galactose^31,32^ — their rates of proliferation are lower and their rates of death are higher^33^. As such, we predicted that galactose would create an environment unfavourable to cells with nonsynonymous heteroplasmy, but not synonymous heteroplasmy. Conversely, if heteroplasmic shifting is caused by drift, we would expect the same heteroplasmy dynamics in cells regardless of edit type.

In bulk analysis, we observed a strong selection against the nonsynonymous variant. We found that this effect was present in glucose and was accentuated in galactose (initial missense heteroplasmy in highly heteroplasmic cells: 65.55 ± 1.10%; day 5: 56.87 ± 0.78% in glucose *P* < 0.001 versus 38.33 ± 4.4% in galactose *P* < 0.001 (Fig. 3c). Conversely, we did not observe selection against the synonymous variant in either culture condition (initial silent heteroplasmy in highly heteroplasmic cells: 69.59 ± 0.46%; day 5: 69.79 ± 0.37% in glucose *P* > 0.05 versus 69.07 ± 1.24% in galactose *P* > 0.05) (Fig. 3d). The fact that heteroplasmic shifting occurs for the nonsynonymous but not the synonymous variant argues against simple drift in our system and implies selection.

## Differential cell fitness of heteroplasmic cells

We next sought to determine whether the apparent selection was acting at the level of the cell or at the intracellular level. We transfected cells with LHON or SILENT editors, sorted for edited cells with either high or low expression of DdCBEs, and measured their fitness in culture. We did not observe any fitness defect in LHON-edited or SILENT-edited cells with low heteroplasmy levels (population doublings: LHON 1.28 ± 0.18, SILENT 1.22 ± 0.04; *P* > 0.05; dead cells (%): LHON 3.27 ± 2.12, SILENT 3.27 ± 1.07; *P* > 0.05) (Fig. 3e). However, in the high heteroplasmy populations, we saw that LHON-edited cells grow slower than SILENT-edited cells (population doublings per day: SILENT 1.3 ± 0.18, LHON 1.00 ± 0.16; *P* < 0.001), consistent with a fitness defect for the LHON-edited population. Cell viability was the same in both populations (dead cells (%): SILENT 2.23 ± 0.51, LHON 2.27 ± 0.31; *P* > 0.05) (Fig. 3f). Furthermore, this fitness defect was exaggerated when we cultured cells in galactose; we observed a strong growth defect and massive cell death in the LHON-edited cells, but not in the SILENT-edited cells (population doublings per day: LHON −0.64 ± 0.11, SILENT 0.52 ± 0.07; *P* < 0.001; dead cells (%): LHON 35.93 ± 2.5, SILENT 7.63 ± 0.71; *P* < 0.001) (Fig. 3f).

**Extended Data Fig. 3:**
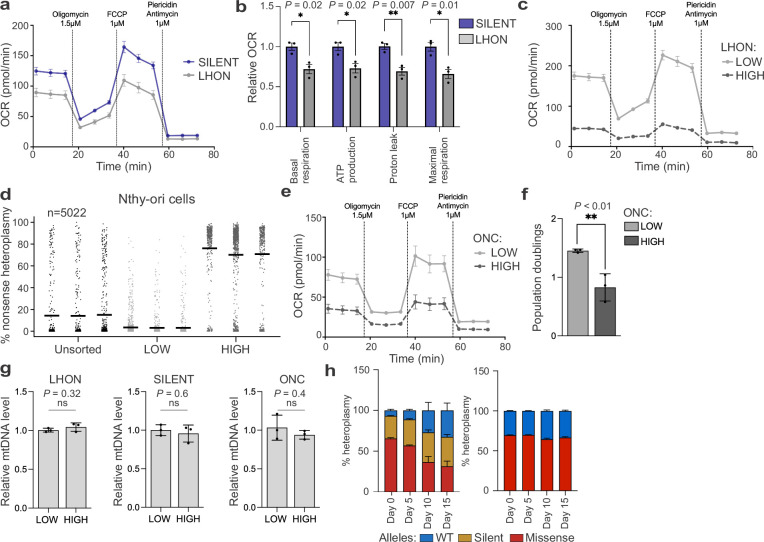
Characterization of heteroplasmic cells. **a**, Oxygen consumption rate (OCR) in LHON- and SILENT-edited cells for *n* = 3 biological replicates. Error bars reflect the mean ± s.e.m. and **b**, relative OCR values in LHON- and SILENT-edited cells for each of the segments in **a** that characterize different respiratory parameters. Data for *n* = 3 independent biological replicates are shown. Error bars reflect the mean ± s.d. *P < 0.05; **P < 0.01; by Student’s unpaired two-tailed t-test. FCCP, carbonyl cyanide-4-(trifluoromethoxy)phenylhydrazone. **c**, OCR in LHON-edited cells with low and high levels of missense heteroplasmy, *n* = 3 independent biological replicates. Error bars reflect the mean ± s.e.m. **d**, Single cell heteroplasmy measured by SCI-LITE in Nthy-ori cells edited with oncocytoma (ONC) editor and sorted for low and high levels of editor expression, *n* = 3 biological replicates of edited cells are shown. Lines represent the mean for single biological replicates. Dots represent single cells. **e**, OCR in edited Nthy-ori cells with low and high levels of nonsense heteroplasmy, *n* = 3 independent biological replicates. Error bars reflect the mean ± s.e.m. **f**, Population doublings in ONC-edited Nthy-ori cells with low and high levels of nonsense heteroplasmy, *n* = 3 independent biological replicates. Error bars reflect the mean ± s.d. **P < 0.01; by Student’s unpaired two-tailed t-test. **g**, Relative mtDNA levels in low and high heteroplasmic 293 T cells edited with LHON, SILENT and ONC DdCBE measured by qPCR, *n* = 3 independent biological replicates. Error bars reflect the mean ± s.d. ns > 0.05; by Student’s unpaired two-tailed t-test **h**, Bulk heteroplasmy measurement in cells treated with LHON or SILENT editors showing mean frequency of each allele, *n* = 3 biological replicates. Error bars reflect the mean ± s.d. Source Data

To determine whether the growth defect that we observed is due to the complex I deficiency, we compared respiration rates of LHON-edited and SILENT-edited cells. We observed that LHON-edited cells have lower rates of respiration (Extended Data Fig. 3a,b) than the SILENT-edited cells, consistent with a complex I defect caused by the *MT-ND4* missense mutation. Moreover, we observed that LHON-edited cells with high heteroplasmy have lower rates of respiration than cells with low heteroplasmy (Extended Data Fig. 3c). To investigate whether this effect extends to other cell lines and mtDNA variants, we transfected SV40-immortalized normal human thyroid follicular epithelial cells (Nthy-ori 3-1)^33,34^ with our ONC DdCBE, and then we sorted for cells with either high or low DdCBE expression. SCI-LITE analysis revealed the expected concordance between heteroplasmy levels and editor expression (Extended Data Fig. 3d). We observed that edited cells with high heteroplasmy have lower rates of respiration than cells with low heteroplasmy (Extended Data Fig. 3e), and cells with high heteroplasmy consistently grow slower than cells with low heteroplasmy (Extended Data Fig. 3f). For all three editors (LHON, SILENT and ONC), we did not observe differences in mtDNA levels between low and high heteroplasmic cells (Extended Data Fig. 3g). Together, these results show that cells with lower nonsynonymous heteroplasmy levels have a cell fitness advantage because of the higher proliferation rates.

## Joint distribution of silent and missense heteroplasmy

Next, we used SCI-LITE to track multiple alleles co-occurring within individual cells. We isolated highly heteroplasmic LHON-edited or SILENT-edited cells using FACS, cultured them and collected samples for SCI-LITE at days 0, 5, 10 and 15 of culture (Fig. 3g). After 5 days, we observed a decrease in the missense heteroplasmy in cells treated with LHON DdCBE (Kolmogorov–Smirnov *D* = 0.29, *P* < 0.001; median day 0 = 0.73, *n* = 1,090; median day 5 = 0.62, *n* = 993, Mann–Whitney *P* < 0.001), and this effect was accentuated at day 10 (Kolmogorov–Smirnov *D* = 0.53, *P* < 0.001; median day 10 = 0.51, *n* = 1,455, Mann–Whitney *P* < 0.001) and day 15 (Kolmogorov–Smirnov *D* = 0.56, *P* < 0.001; median day 15 = 0.48, *n* = 2,991, Mann–Whitney *P* < 0.001) (Fig. 3h). Consistent with the missense heteroplasmy leading to differential cell proliferation rates, the decrease of missense heteroplasmy was associated with an increase of silent heteroplasmy in this same LHON-edited population (Kolmogorov–Smirnov at day 5 *D* = 0.20, *P* < 0.001; Kolmogorov–Smirnov at day 10 *D* = 0.36, *P* < 0.001; Kolmogorov–Smirnov at day 15 *D* = 0.36, *P* < 0.001; median day 0 = 0.24, *n* = 1,090; median day 5 = 0.32, *n* = 993; median day 10 = 0.39, *n* = 1,455; median day 15 = 0.40, *n* = 2,991, for day 0 versus day 5, day 0 versus day 10 and day 0 versus day 15 Mann–Whitney *P* < 0.001) (Fig. 3h). At the same time, we did not observe any shift in the silent heteroplasmy distribution in cells treated with SILENT DdCBE (Kolmogorov–Smirnov at day 5 *D* = 0.03, *P* > 0.05; Kolmogorov–Smirnov at day 10 *D* = 0.05, *P* > 0.05; Kolmogorov–Smirnov at day 15 *D* = 0.03, *P* > 0.05; median day 0 = 0.77, *n* = 1,600; median day 5 = 0.77, *n* = 3,093; median day 10 = 0.77, *n* = 1,573; median day 15 = 0.77, *n* = 2,283, for day 0 versus day 5 Mann–Whitney *P* = 0.45, for day 0 versus day 10 Mann–Whitney *P* = 0.19, for day 0 versus day 15 Mann–Whitney *P* = 0.90) (Fig. 3i). These SCI-LITE results are consistent with our bulk assays (Extended Data Fig. 3h), further confirming that heteroplasmic shifting occurs only in the context of the nonsynonymous mutation.

**Extended Data Fig. 4:**
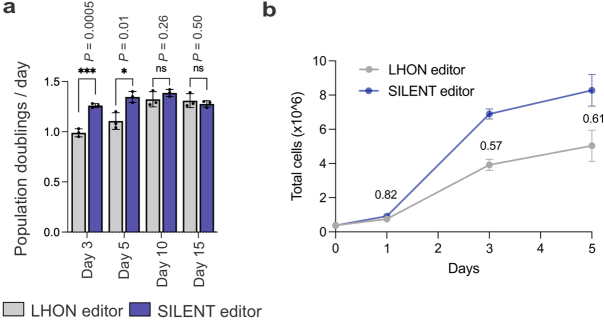
Cells with high levels of missense heteroplasmy have a growth disadvantage in standard cell culture conditions. Population doublings per day (**a**) and cumulative cell counts (**b**) in LHON- and SILENT-edited high heteroplasmic cells. In **b**, numbers reflect the fraction of total LHON-edited cells vs SILENT-edited cells. Data for *n* = 3 independent biological replicates are shown. Error bars reflect the mean ± s.d. *P ≤ 0.05; ***P ≤ 0.001; ns > 0.05; by Student’s unpaired two-tailed t-test.

In the LHON-edited population, culturing cells for 5 and 10 days caused a population shift from high missense and low silent heteroplasmic cells towards low missense and high silent heteroplasmic cells, and we observed a more modest change between the 10-day and 15-day timepoints. The current dogma proposes a ‘heteroplasmic threshold’ in which heteroplasmy below a certain level, typically 60–90%, may not yield a strong phenotype^1,35^. By day 10, the majority of cells (70.6 ± 0.18%) already had missense heteroplasmy below 60% (Fig. 3g), and when we compared growth rates of LHON-edited and SILENT-edited cells, we observed a growth defect of LHON-edited cells at day 3 and day 5, but not at day 10 and day 15 (Extended Data Fig. 4), suggesting that by the later timepoints, the mutant heteroplasmy had dropped below the heteroplasmic threshold.

**Extended Data Fig. 5:**
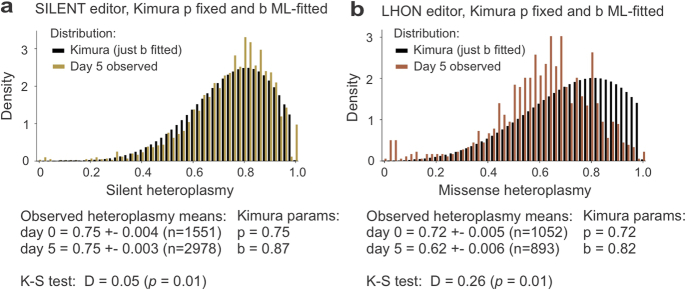
Kimura distribution fitting. Density histograms showing the distribution of observed heteroplasmy in SILENT-edited (**a**) or LHON-edited (**b**) cells, and their corresponding maximum likelihood (ML) fits to the two-parameter Kimura(p,b) distribution after fixing the initial heteroplasmy parameter, p, to be equal to the experimentally-observed day 0 heteroplasmy. For the LHON-edited cells, the large shift in mean heteroplasmy between day 0 and day 5 cannot be accounted for by fitting only the Kimura drift parameter, b, and yields a poor fit (K-S D = 0.26) in contrast to the superior fit observed for the SILENT-edited cells (K-S D = 0.05). Source Data

**Extended Data Fig. 6:**
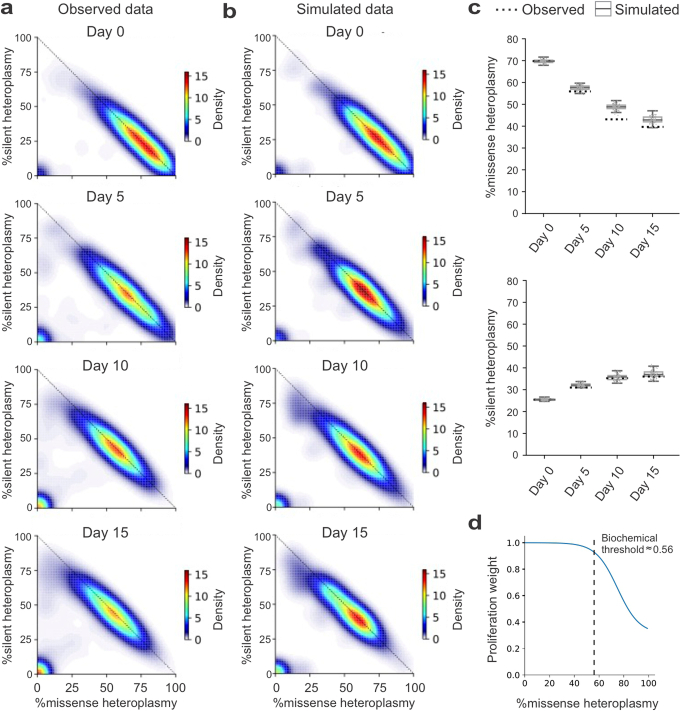
Decline in nonsynonymous mtDNA heteroplasmy can be largely explained by modeling selection at the level of cell fitness. We modeled heteroplasmy dynamics in LHON-edited cells under the assumption that the relative probability of a cell dividing, compared to any other cell, is entirely determined by its missense heteroplasmy level, with no intracellular effects. The model was fit based on the LHON-edited SCI-LITE time course, in which cells were grown for 5, 10 and 15 days. **a**-**b**, 2D kernel density estimate plots showing the joint missense and silent heteroplasmy distribution in observed data of the LHON-edited cells (**a**) and in simulated data based on our modeling (**b**). **c**, Distribution of mean heteroplasmy estimates from n = 100 simulated experiments (box plots) compared to the observed mean heteroplasmy based on SCI-LITE data (dotted horizontal lines). The boxes show the interquartile range, the line through the box shows the median, the whiskers show 1.5 * interquartile range. **d**, The chance of a cell dividing on each simulated day of the experiment was weighted by an inverse sigmoid function of the cell’s missense heteroplasmy. The fitted inverse sigmoid function model corresponds to a threshold at ~56% heteroplasmy. Source Data

**Extended Data Fig. 7:**
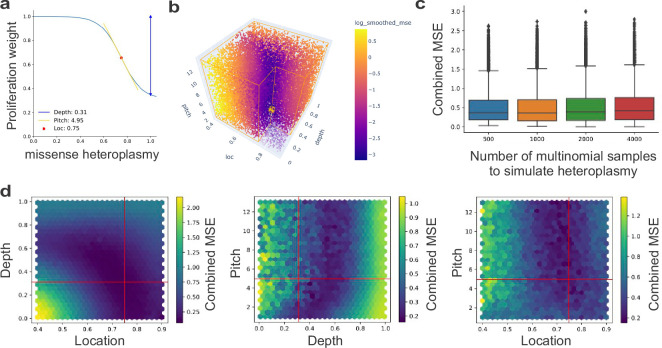
Parameter search for computer model of selection acting at level of cell fitness. **a**, Fitting the three parameters (location, depth, and pitch) of the inverse sigmoid function resulted in the function shown. **b**, 3D scatter plot showing the 20,000 models we trained to fit the inverse sigmoid to our data. Each dot represents a model trained with a random combination of the three parameters, and the color indicates the log-transformed smoothed and combined mean squared error (see Methods for details) of the resulting fit to our observed data. The minimum MSE is indicated by a large gold-colored marker. **c**, A range of multinomial sample sizes per cell for simulating the heteroplasmy was tested, and 1000 was chosen. The boxplots represent a total of n = 20,000 models. The 500 box represents 5071 models, the 1000 represents 4979 models, the 2000 represents 4944 models, and the 4000 represents 5006 models. The boxes show the interquartile range, the line through the box shows the median, the whiskers show 1.5 * interquartile range. **d**, Plots showing the marginal 2D facets of the 3D plot in panel **b**. Each hexagon is colored based on the mean smoothed and combined MSE for models in that 2D bin. Red lines indicate the chosen optimal parameter combination.

We then asked whether the heteroplasmy shifts that we observed in LHON-edited or SILENT-edited cells can be explained by a simple model of drift based on the ‘Kimura’ distribution^36,37^. We found that the maximum likelihood fit of the Kimura distribution to our observed silent heteroplasmy distribution was very good (Extended Data Fig. 5a). By contrast, the fit of the Kimura distribution to our cells with missense heteroplasmy was qualitatively poor (Extended Data Fig. 5b), consistent with the notion that the missense heteroplasmy shift cannot be explained by simple random drift. Moreover, we found that the joint distribution of missense and silent heteroplasmy could be explained by a computer model that assumes that cell fitness is simply a function of the missense heteroplasmy level (see Methods for details) (Extended Data Figs. 6 and 7). Our model produced simulated results that were very similar to our observed data (Extended Data Fig. 6a–c) and it suggested that the heteroplasmy threshold in our system is about 56% (Extended Data Fig. 6d). At the 65.6% mean heteroplasmy level that we observed in the LHON-edited cells at day 0 (Fig. 3c), our model predicts an 18% lower doubling rate per day for LHON-edited cells than for SILENT-edited or wild-type cells; this approximates what we observed in our empirical measurements, in which the LHON-edited population showed a 21% lower doubling rate per day than the SILENT-edited population (Fig. 3f).

## Selection acts at the level of cell fitness

**Fig. 4:**
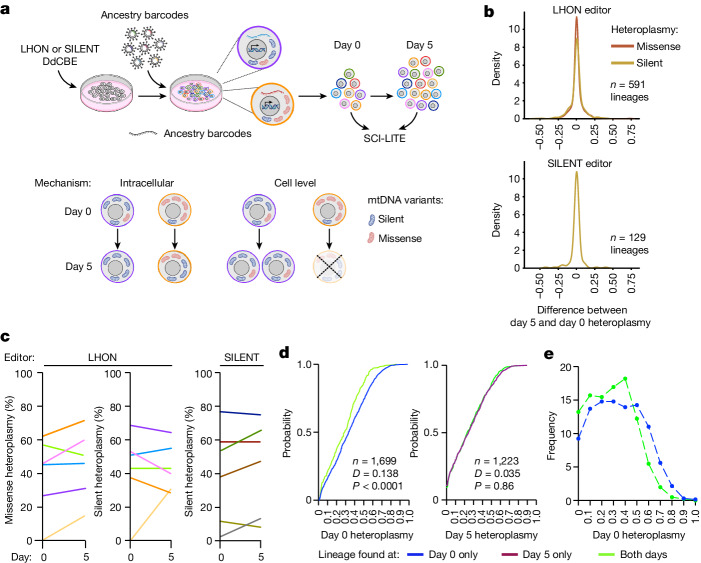
Selection against nonsynonymous mtDNA variants occurs at the level of cell fitness. **a**, Schematic overview of the lineage tracing experiment. 293T cells were transfected with LHON or SILENT DdCBEs and subsequently transduced with a lentiviral library with unique ancestry barcodes so that each heteroplasmic cell expressed a single, unique ancestry barcode. Ancestral lineages were expanded, cells were harvested at day 0 and day 5, and multiplexed SCI-LITE was performed to capture mtDNA heteroplasmy and ancestry barcodes. Schematics in part **a** were creating using BioRender (https://biorender.com). **b**, Distribution of differences in heteroplasmy levels between day 5 and day 0, for all ancestral lineages that were detected at both days. **c**, Heteroplasmy levels in randomly selected ancestral lineages at day 0 and day 5. Each line represents one unique ancestral lineage and visualizes the mean heteroplasmy level at each day. See Extended Data Fig. 8 for all ancestral lineages. **d**, Single-cell heteroplasmy in LHON-edited cells. The graphs show empirical cumulative distributions of missense heteroplasmy in ancestry lineages found at only one timepoint or at both timepoints. The Kolmogorov–Smirnov test was used to calculate *D* statistics and *P* values. **e**, Relative frequency of binned missense heteroplasmy in ancestral lineages found at day 0 only or at both timepoints. Cells with ancestry barcodes that are found only at day 0 have significantly higher missense heteroplasmy by Kolmogorov–Smirnov test, suggesting cell-level selection against these lineages. Source Data

To directly validate that selection operates at the level of cell fitness, we performed a multiplexed SCI-LITE experiment in which we simultaneously monitored single-cell heteroplasmy and cell lineage. We transfected cells with LHON or SILENT DdCBEs and subsequently transduced edited cells with a lentiviral library with unique ancestry barcodes so that each edited cell expressed a single, unique ancestry barcode (Fig. 4a). We then performed SCI-LITE to capture both mtDNA heteroplasmy and ancestry barcodes together in single cells at different timepoints (Fig. 4a). If cell-level selection drives purification of the missense mtDNA variant, we would expect little change in heteroplasmy within ancestry lineages, whereas lineages with high heteroplasmy will drop out in the later timepoint due to their growth defect. Conversely, if intracellular selection drives purification of the missense variant, then we would expect a systematic decrease in heteroplasmy over time in all ancestry lineages (Fig. 4a).

**Extended Data Fig. 8:**
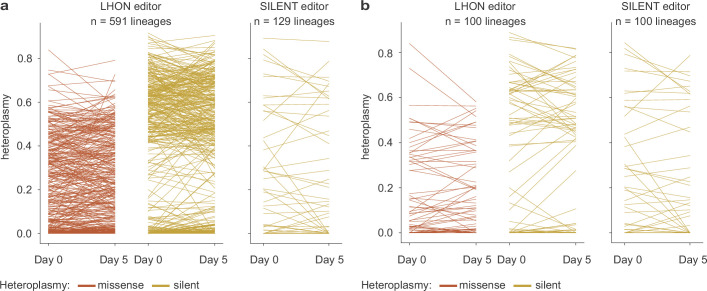
Observed heteroplasmy in ancestral lineages. **a**-**b**, Heteroplasmy levels in all 720 ancestral lineage with cells detected at both days (**a**), and a random subsample of 100 of those lineages (**b**), in LHON-edited and SILENT-edited populations at day 0 and day 5. Each line segment represents a unique ancestral lineage and connects that lineage’s mean heteroplasmy at day 0 with that of day 5.

We observed that, on average, heteroplasmy levels were stable in the ancestry lineages over time, and the distribution of heteroplasmy differences between day 5 and day 0 centred around zero (Fig. 4b). Our observations were extremely similar for missense and silent variants (Fig. 4c and Extended Data Fig. 8). Our results show that, typically, heteroplasmy is stable within lineages and suggests only minimal contribution of intracellular selection.

We also observed that some lineages with high missense heteroplasmy dropped out between the early and late timepoints. LHON-edited cells with ancestry barcodes detected only at day 0 had significantly higher missense heteroplasmy than cells with ancestry barcodes detected at both timepoints (Kolmogorov–Smirnov *D* = 0.138, *P* < 0.0001; median day 0 only = 0.33, *n* = 1,298; median of both timepoints = 0.29, *n* = 401, Mann–Whitney *P* < 0.001) (Fig. 4d). Similarly, LHON-edited cells with ancestry barcodes detected at both timepoints had significantly higher silent heteroplasmy than cells with ancestry barcodes found only at day 0 (Kolmogorov–Smirnov *D* = 0.129, *P* < 0.0001; median day 0 only = 0.55, *n* = 1,298; median of both timepoints = 0.59, *n* = 401, Mann–Whitney *P* = 0.003). LHON-edited cells with ancestry barcodes detected only at day 5 had missense heteroplasmy comparable with cells with ancestry barcodes found at both timepoints (Kolmogorov–Smirnov *D* = 0.035, *P* = 0.86; median day 5 only = 0.24, *n* = 907; median of both timepoints = 0.23, *n* = 457, Mann–Whitney *P* = 0.77). Looking more closely at the LHON-edited lineages, we found that lineages with heteroplasmy exceeding approximately 60% experienced dropout between day 0 and day 5 (Fig. 4e). This observed threshold level is close to the 56% threshold identified by our computer modelling (Extended Data Fig. 6d).

## Sign of fitness effect is environment dependent

Our above studies demonstrate that the effect of mtDNA heteroplasmy on cell fitness is a major determinant of the heteroplasmic shifting that we observe in dividing cells. If this is true, then, in principle, even though we have so far only observed negative selection against the nonsynonymous alleles, we ought to be able to positively select for these alleles if we can find environments in which defective OXPHOS confers a cell fitness advantage. In fact, we have previously identified ‘environments’ in which loss of biochemical complex I activity is beneficial. Therefore, we tested whether we could actively select for truncating complex I mtDNA heteroplasmy by placing cells in such environments.

**Fig. 5:**
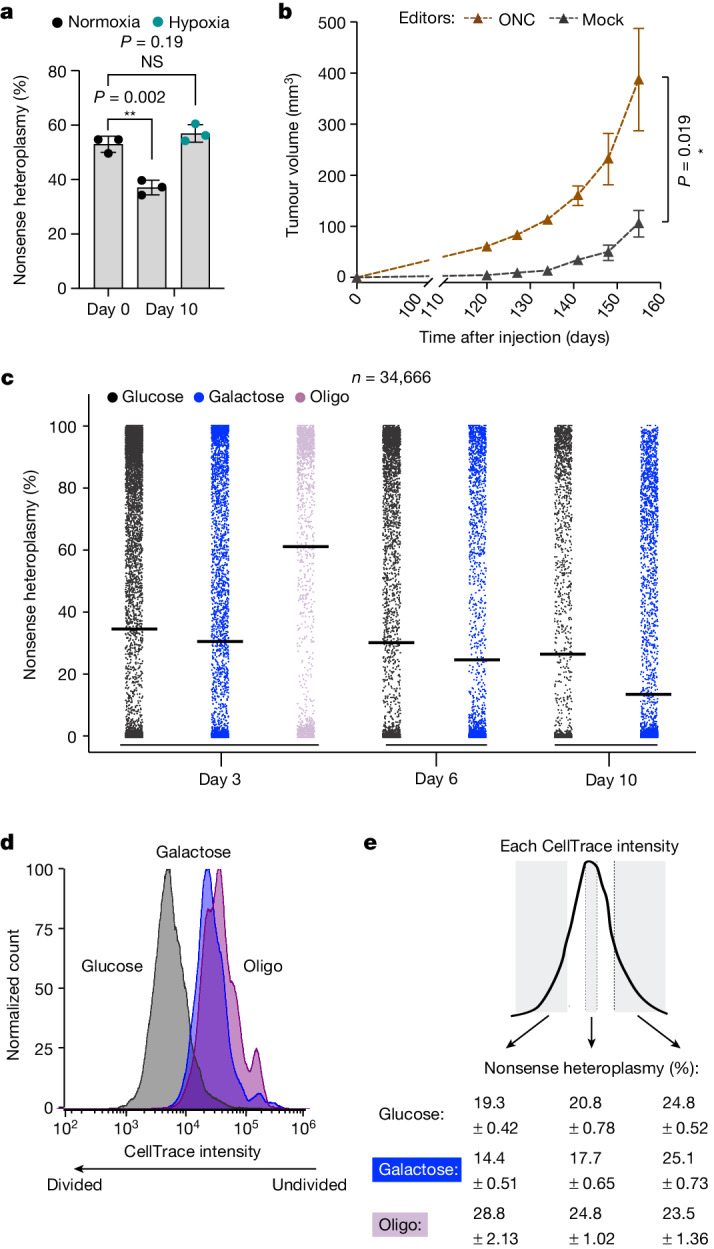
A truncating complex I mtDNA mutation can be harmful, neutral or even beneficial to cell fitness depending on the environment. **a**, Bulk mtDNA heteroplasmy in 293T cells transfected with ONC DdCBEs. Error bars represent the mean ± s.d. for *n* = 3 biological replicates. **P < 0.01 and NS > 0.05, by Student’s unpaired two-tailed *t*-test. **b**, Nthy-ori cells were edited with an active (ONC) or inactive (mock) DdCBE and 2 × 10^6^ cells were implanted into immunodeficient mice with an equal volume of Matrigel (50 μl). Tumour volume measurements of xenografts are shown. Error bars indicate mean ± s.e.m.; *n* = 5; *P < 0.05, by Mann–Whitney unpaired two-tailed test. **c**, Single-cell heteroplasmy in ONC-edited cells interrogated using SCI-LITE for *n* = 3 biological replicates and 34,666 single cells. See Extended Data Fig. 9b for a strip plot showing single replicates. **d**,**e**, CellTrace analysis. K562 cells edited with the ONC DdCBE were stained with a fluorescent dye allowing for tracing of cell divisions. Cells with higher CellTrace stain intensity underwent fewer divisions than cells with lower CellTrace stain intensity (**d**). Cells were stained with CellTrace and cultured for 4 days in media containing glucose, galactose or oligomycin, then harvested and sorted based on CellTrace intensity (**e**). Heteroplasmy levels were measured by bulk amplicon sequencing in sorted populations, and these measurements revealed decreased (galactose) or increased (oligomycin) heteroplasmy levels in the proliferating cells. Data for *n* = 3 biological replicates are shown. Source Data

First, we considered growth in normoxia (21% ambient O_2_) versus hypoxia (1% ambient O_2_), as we have previously shown that defective complex I can be buffered by ambient hypoxia^38^. Bulk heteroplasmy analysis of 293T cells edited with the ONC DdCBE revealed that culturing cells for 10 days in normoxia caused a decrease in mean heteroplasmy, whereas culturing cells in hypoxia preserved their initial heteroplasmy level (53 ± 2.94% initial heteroplasmy; 37.10 ± 2.7% mean heteroplasmy in normoxia after 10 days *P* < 0.01 versus 56.97 ± 3.22% mean heteroplasmy in hypoxia after 10 days *P* > 0.05) (Fig. 5a).

Second, in Hürthle cell carcinoma of the thyroid, complex I mtDNA mutations have been proposed to be under positive selection and contribute to tumorigenesis^8^. This is based on the observation that joint nuclear and mtDNA sequencing identifies disruptive, complex I mtDNA mutations as some of the earliest genetic features of these tumours, which go on to accrue high levels of mutant heteroplasmy. One such mutation is the variant introduced by the ONC DdCBE^8^. To directly test whether this nonsynonymous mtDNA mutation can be beneficial to cell fitness in vivo and promote tumour growth, we treated SV40-immortalized normal human thyroid epithelial cells (Nthy-ori 3-1) with the ONC DdCBE, and then subcutaneously implanted the edited cells into immunodeficient mice. As a control, we used a catalytically dead ONC DdCBE targeting the same mtDNA site^4^. As expected, we did not observe editing using the dead ONC DdCBE, but we obtained a range of heteroplasmy values in the cells edited with the active ONC DdCBE (Extended Data Fig. 3d). We monitored the mice for tumour growth and found that introducing this oncocytoma-related truncating complex I mutation significantly accelerated tumour formation (Mann–Whitney *P* = 0.019) (Fig. 5b).

**Extended Data Fig. 9:**
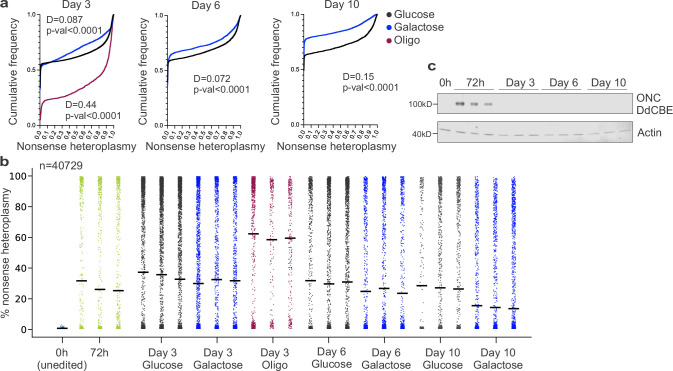
Environment dictates the sign of mtDNA selection. **a**, Single cell heteroplasmy in ONC-edited cells interrogated using SCI-LITE. Graph shows cumulative distributions of *MT-ND4* nonsense heteroplasmy for *n* = 3 biological replicates. Number of cells at each day: Day 3: *n* = 16,544; Day 6: *n* = 8,770; Day 10: *n* = 9,352. Kolmogorov–Smirnov test was used to calculate *D* statistics and *P* value. **b**, Single-cell heteroplasmy in ONC-edited cells interrogated using SCI-LITE. Graph shows single replicates from main Fig. 5c. Data for *n* = 3 biological replicates of edited cells are shown. Lines represent the mean for single biological replicates. Dots represent single cells. **c**, Western blots of ONC DdCBE showing expression of FLAG-tagged DdCBE half. Data for *n* = 3 biological replicates are shown.

Third, we have previously shown that in the presence of the complex V inhibitor oligomycin, simultaneous loss of complex I activity (either via chemical poisoning of complex I or by knockout of a nuclear-encoded subunit) would confer a benefit to cell fitness^39^. Thus, we hypothesized that oligomycin would create conditions that lead to active, positive selection of mutant mtDNA heteroplasmy. We compared growth in glucose, galactose and oligomycin, and performed SCI-LITE to assess heteroplasmy. Culturing ONC-edited 293T cells for 3 days in the presence of galactose versus glucose caused a change in the shape of the heteroplasmy distribution (Kolmogorov–Smirnov *D* = 0.087, *P* < 0.001; median glucose day 3 = 0.01, *n* = 12,185; median galactose day 3 = 0.01, *n* = 5,501, Mann–Whitney *P* = 0.50). By contrast, culturing cells for 3 days in the presence of oligomycin caused a marked shift in heteroplasmy distribution towards mutant mtDNA (Kolmogorov–Smirnov *D* = 0.44, *P* < 0.001; median glucose day 3 = 0.01, *n* = 12,185; median oligo day 3 = 0.87, *n* = 1,626, Mann–Whitney *P* < 0.001). Further culturing the cells for 6 and 10 days in glucose or galactose resulted in a time-dependent shift in the heteroplasmy distribution towards wild-type mtDNA in both conditions, although this shift was accelerated in galactose (day 6: Kolmogorov–Smirnov *D* = 0.072, *P* < 0.001; day 10: Kolmogorov–Smirnov *D* = 0.15, *P* < 0.001; median glucose day 10 = 0, *n* = 3,621; median galactose day 10 = 0, *n* = 7,503, Mann–Whitney *P* < 0.001) (Fig. 5c and Extended Data Fig. 9a). We obtained very similar results across biological replicates (Extended Data Fig. 9b). Of note, DdCBE expression cannot be detected by western blot 6 days post-transfection (day 3 of treatment); therefore, the changes in the heteroplasmy levels that we observed are due to selection rather than editing (Extended Data Fig. 9c and Supplementary Fig. 3).

To further confirm that the mechanism is occurring via differences in cell fitness, we performed a CellTrace analysis and measured heteroplasmy following different numbers of cell doublings^40,41^. We used human K562 cells, which we engineered to express the ONC DdCBE in an inducible manner. Cells were treated with doxycycline for 3 days to induce editing (editors on) and then doxycycline was washed away to turn off the editors (editors off). Heteroplasmic K562 cells (editors off) were stained with CellTrace dye to monitor cell divisions in vivo by dye dilution, and cultured for 4 days in media containing glucose, galactose or oligomycin (Fig. 5d). For the glucose and galactose conditions, we observed that the cells with lower dye intensity, which divided more times, had lower heteroplasmy than the cells with higher dye intensity, which divided fewer times (heteroplasmy difference in glucose: 5.5 ± 0.91%, *P* < 0.001; in galactose: 10.77 ± 0.65%, *P* < 0.001) (Fig. 5e). By contrast, cells treated with oligomycin that divided more times had higher heteroplasmy than cells that divided fewer times (heteroplasmy difference: 5.28 ± 2.72%, *P* < 0.05). Of note, galactose and oligomycin led to comparable effects on cell growth based on the CellTrace intensity (Fig. 5d), yet selected for cells on opposite ends of the heteroplasmy distribution. Therefore, our results indicate that selection at the level of cell fitness towards the ‘beneficial allele’ depends on the environment.

## Discussion

We provide multiple lines of evidence that classic heteroplasmic shifting observed in dividing cells is governed by selection (not simple drift) that acts at the level of cell fitness (and not intracellularly). First, in standard culture conditions, cell populations purge nonsynonymous mtDNA variants, whereas synonymous variants are maintained. Second, cells with a high fraction of silent heteroplasmy have a cell fitness advantage over cells with a high nonsynonymous heteroplasmy. Third, in a population of cells with both missense and silent heteroplasmic mtDNA variants, we observed a time-dependent shift in the joint heteroplasmy distribution of both variants that could be modelled using a simple simulation of selection acting at the level of cell fitness without invoking intracellular effects. Fourth, lineage tracing revealed that heteroplasmy remains stable in individual cell lineages, even though the overall heteroplasmy of the cell population undergoes a dynamic shift. Fifth, we found that galactose and oligomycin lead to comparable deficits in cell growth; however, in galactose, the cells that divide the most have the lowest nonsynonymous heteroplasmy, whereas in oligomycin, cells that divide the most have the highest nonsynonymous heteroplasmy. Our work demonstrates that a given nonsynonymous heteroplasmy can be harmful, neutral or even beneficial to cell fitness, but that the ‘sign’ of the effect is wholly dependent on the environment. Although ‘sign epistasis’ is widely appreciated in classical gene–gene interactions, this concept has not been extended to mtDNA heteroplasmy, especially with respect to environment.

There is little doubt that maternally inherited mtDNA mutations are causal for diseases such as MELAS (Mitochondrial Encephalopathy, Lactic Acidosis, and Stroke-like episodes) and MERRF (Myoclonic Epilepsy with Ragged Red Fibers) by disrupting OXPHOS^42,43^. However, nonsynonymous mtDNA mutations — especially those impacting complex I — are often reported to accumulate in ageing and in age-associated diseases. It is generally assumed that the nonsynonymous mtDNA mutations disrupt complex I biochemical activity, which then causes bioenergetic or redox disturbances that contribute causally to pathology. Although this is certainly possible, our current work suggests an alternative interpretation that should also be considered: namely, that ageing and other conditions may create an environment in which defective complex I is beneficial to cell fitness, and hence advantageous to cells to accumulate nonsynonymous mtDNA heteroplasmy.

## Methods

### Figures

The following figures were created with BioRender (agreement number QJ26EVEXW9; https://biorender.com): Figs. 1a, 2a,d,e and 4a. The following figures were created using GraphPad Prism (license number 27313 to MGB; https://graphpad.com): Figs. 1f, 3c,d,e,f, 3h,i, 4c,d,e, 5a,b; Extended Data Figs. 2b, 3a,b,c, 3e,f,g,h, 5a,b, 9a.

### Reagents

All oligonucleotides and reagents are listed in Supplementary Tables 1–3.

### Mammalian cell culture

293T (ATCC CRL-3216), HeLa (ATCC CCL-2) and K562 (ATCC CCL-243) cells were purchased from the ATCC (American Type Culture Collection). Nthy-ori 3-1 cells were purchased from the European Collection of Cell Cultures (ECACC) (90011609). Cell lines were authenticated by STR profiling by the supplier. 293T, HeLa and K562 cells were routinely cultured in DMEM with high glucose and pyruvate (Gibco), supplemented with 10% (v/v) FBS (Gibco). Nthy-ori 3-1 cells were routinely cultured in RPMI 1640, supplemented with 10% (v/v) FBS (Gibco). Cell cultures were maintained at 37 °C with 5% CO_2_ and were tested negative for mycoplasma. For selected experiments described in the main text, cells were cultured in DMEM without glucose (Gibco), supplemented with 10% (v/v) dialysed FBS (Gibco), 25 mM glucose or galactose (Sigma), 1 mM sodium pyruvate (Gibco) and 50 μg ml^−1^ uridine (Sigma). In the indicated experiments, cells were cultured in the presence of 2.5 nM oligomycin A (Sigma). In the indicated experiments, cells were cultured in the presence of 100 ng ml^−1^ EtBr (Sigma). For selected experiments, described in the main text, cell cultures were maintained in a hypoxia chamber glove box at 37 °C with 1% O_2_.

### Proliferation and viability experiments

Cells were seeded in triplicate at 0.5 × 10^6^ (glucose conditions) or 2 × 10^6^ (galactose or oligomycin conditions) in 2 ml media in 6-well plates, and cell counts were performed after 3 days of culture using the Vi-Cell XR Cell Viability Analyzer (Beckman Coulter). Population doubling was calculated as log_2_(final density/seeding density). Cell viability was calculated based on trypan blue staining performed with the Vi-Cell XR Cell Viability Analyzer (Beckman Coulter).

### Transient transfection of 293T cells

Cells were seeded in triplicate at 0.5 × 10^6^ in 2 ml media in 6-well plates, 24 h before transfection. Lipofection was performed at a cell density of approximately 50%. Cells were transfected with a total of 1,000 ng of plasmid DNA (500 ng of each DdCBE monomer), using 3 μl Lipofectamine 2000 reagent (Thermo Fisher Scientific) per well. Cells were split 24 h after lipofection and used for experiments. See Supplementary Table 2 for a list of plasmids used for transfection.

### Transient transfection of Nthy-ori 3-1 cells

Cells were transfected using the SF cell line 4D-nucleofector X kit (Lonza) with the CN-114 program according to the manufacturer’s protocol. One million cells were transfected with a total of 2,000 ng of plasmid DNA (1,000 ng of each DdCBE monomer). Cells were split 24 h after transfection and used for experiments. See Supplementary Table 2 for a list of plasmids used for transfection.

### Cell tracing

K562 cells were stained with CellTrace Far Red Cell Proliferation reagent according to the manufacturer’s protocol. Cells were then cultured for 4 days and used for FACS.

### Lineage tracing

293T cells were edited using LHON or SILENT DdCBEs according to the transient transfection protocol described above. Twenty-four hours after transfection, cells were transduced with a CloneTracker lentiviral barcode library (Cellecta) containing 10 million unique barcodes, enabling clonal expansion tracking. Cells were transduced at a multiplicity of infection (MOI) of 0.1 to ensure that each cell had one unique, expressed barcode. Infections were performed using 2 million cells in 10-cm culture dishes, and in media supplemented with 6 μg ml^−1^ polybrene. Twenty-four hours after transduction, cells were selected for 48 h with 2 μg ml^−1^ puromycin (Invitrogen). At that point, cells were counted and split 1/20 to decrease diversity of barcodes. Cultures were expanded for 3 days, and approximately 15% of cells were removed for SCI-LITE. The remaining cells were plated, and cultures were expanded for an additional 5 days and collected for SCI-LITE. SCI-LITE was performed to capture both the *MT-ND4* transcript and the expressed lineage barcodes according to the detailed protocol, with the difference that PCRs to amplify *MT-ND4* and lineage barcodes were performed separately, and all PCR products were pooled together after the PCR purification step.

### SCI-LITE time course experiments design

For the LHON and SILENT SCI-LITE time course (Fig. 3g–i), the population size and splitting strategy (done in biological triplicate) were as follows: on day 0, we removed 1 million cells from approximately 4 million LHON-edited cells and approximately 7 million SILENT-edited cells for SCI-LITE, and seeded the remaining cells for further culture. Cells were passaged at days 3, 5, 8, 10 and 13 at 1:2 ratio for a 2-day interval and at 1:3 ratio for a 3-day interval. At three timepoints (days 5, 10 and 15), 1 million cells were collected for SCI-LITE from each replicate.

For the ONC SCI-LITE time course (Fig. 5c), the population size and splitting strategy (done in biological triplicate) were as follows: on day 0, we seeded 0.5 million cells for glucose and 2 million cells for galactose and oligomycin conditions. On day 3, 0.5 million of 4 million cells were removed for SCI-LITE from each replicate, and the remaining cultures were seeded further for glucose and galactose conditions. All cells were collected for oligomycin conditions, and 0.5 million cells were used for SCI-LITE. On day 6, 0.5 million cells from each replicate were removed for SCI-LITE, and the remaining cultures were seeded further for glucose and galactose conditions. On day 10, 0.5 million cells from each replicate were used for SCI-LITE.

### FACS

Cells transfected with DdCBEs with fluorescent reporters were harvested 24 h after lipofection and used for FACS as described previously^30^. Sorting was performed based on the intensity of eGFP and mCherry fluorescence. See Supplementary Fig. 2d for the representative gating strategy. Cells stained with the CellTrace reagent were harvested 4 days after staining and sorted based on the CellTrace intensity (AF647 channel). SH800S software version 2.1.6 and FCS Express software version 7.10.0007 were used to analyse FACS data.

### Western blot

Cells were harvested, washed with PBS and lysed in 100 μl of ice-cold 1X RIPA buffer (Boston BioProducts) supplemented with protease inhibitor (Roche). Lysates were vortexed with maximum strength for 5 s, followed by incubation on ice for 5 min. Mixing and incubation steps were repeated three times, followed by centrifugation at 12,000*g* for 10 min at 4 °C. 10 µg of protein lysate was resuspended in Laemmli SDS-sample buffer (Boston BioProducts), and was used for gel electrophoresis, performed using Novex Tris-glycine 4–20% gels (Thermo Fisher Scientific), after boiling the lysates for 5 min at 95 °C. Samples were separated by electrophoresis at 200 V for 1 h in Tris-glycine running buffer (Bio-Rad). Semi-dry western blotting was performed using the Trans-Blot Turbo blotting system and nitrocellulose membranes (Bio-Rad). Obtained membranes were blocked in Odyssey Blocking Buffer (LI-COR) for 30 min at room temperature, and incubated with the primary antibodies anti-FLAG (Sigma; 1:1,000 dilution), anti-HA (BioLegend; 1:6,000 dilution) and anti-actin (Cell Signaling; 1:1,000 dilution) in 5% (w/v) BSA (Sigma) in TBST (50 mM Tris-HCl, 150 mM NaCl and 0.05% (v/v) Tween-20, pH 7.4) overnight at 4 °C. Afterwards, membranes were washed three times for 5 min with TBST, and incubated with IRDye-labelled secondary antibodies (goat anti-rabbit 680RD (Li-Cor) or goat anti-mouse 800CW (Li-Cor)) diluted 1:10,000 in 5% (w/v) Blotting-Grade Blocker (Bio-Rad) in TBST for 1 h at room temperature, followed by washing three times for 5 min with TBST. Signals were recorded using an Odyssey Imaging System (Li-Cor).

### Mouse studies

Female NSG (NOD.Cg-Prkdcscid Il2rgtm1Wjl/SzJ) mice of 4–6 weeks of age were purchased from The Jackson Laboratory (RRID: IMSR_JAX:005557) and housed in the animal facility at the Massachusetts General Hospital under ethics oversight from the Massachusetts General Hospital Institutional Animal Care and Use Committee. Housing conditions for the mice were: 12-h dark–light cycle, ambient temperature of 65–75 °F and humidity of 40–60%. Human SV40 immortalized normal thyroid follicular epithelial cells Nthy-ori 3-1 (ref. ^33^) (purchased from the ECACC; cat. 90011609) were nucleofected with either active or catalytically dead (mock) ONC DdCBE, and 2 × 10^6^ cells were injected subcutaneously into the flank of mice (along with 50 μl Matrigel). Tumour size was measured using digital callipers every week, with volumes estimated by the formula volume = ½ × length × width^2^. The maximal tumour size allowed as per Institutional Animal Care and Use Committee guidelines is 1,000 mm^3^, and these limits were not exceeded in any of the experiments. No statistical methods were used to predetermine sample size. The experiments were not randomized, and the investigators were not blinded during experiments or outcome assessment.

### DNA isolation and Sanger sequencing

Total DNA was extracted from cells using the DNeasy Blood & Tissue Kit (Qiagen) according to the manufacturer’s protocol. An *MT-ND4* gene fragment spanning the SNP site in HeLa and 293T cells was amplified with the Phusion Hot Start II High-Fidelity PCR Master Mix (Thermo Fisher Scientific). The primers used for the PCR are listed in Supplementary Table 3. PCR products were purified with the QIAquick PCR Purification Kit (Qiagen) and subjected to Sanger sequencing at Azenta.

### Analysis of relative mtDNA levels by qPCR

qPCRs were performed as previously described^4^. In brief, 4 ng of isolated DNA was used in the qPCR, performed with the use of the iQ SYBR Green Supermix (Bio-Rad) in a 10-μl reaction volume using the CFX Opus 384 machine (Bio-Rad). The relative abundance of the amplified *ND1* gene fragment was normalized to the amplified *B2M* gene fragment.

### RNA isolation and RT–qPCR

Total RNA was extracted from cells with the RNeasy Mini Kit (Qiagen). Isolated RNA (500 ng) was digested with ezDNAse enzyme (Invitrogen) and used for reverse transcription performed with SuperScript IV Reverse Transcriptase (Invitrogen) and 2 μM gene-specific reverse primers. The obtained cDNA was used for qPCR. Analysis of mtRNA abundance was performed with iQ SYBR Green Supermix (Bio-Rad) using the primers listed in Supplementary Table 3.

### Oxygen consumption analysis by Seahorse XF Analyzer

A Seahorse plate was coated with 0.01% (w/v) poly-l-lysine (Sigma), and 1.5 × 10^4^ cells per well were seeded 16 h before analysis with the Seahorse Xfe96 Analyzer (Agilent). Analysis was performed in the Seahorse XF DMEM Medium pH 7.4 (Agilent), supplemented with 10 mM glucose (Agilent), 2 mM l-glutamine (Gibco) and 1 mM sodium pyruvate (Gibco). The Mito stress protocol was applied using 1.5 μM oligomycin, 1 μM FCCP and 1 μM piericidin + 1 μM antimycin. After the run, media was removed from the wells, and cells were stained with Hoechst 33342 (Thermo Fisher Scientific; final 1:5,000 dilution in PBS) for 15 min at room temperature. Next, the staining solution was removed, and cells were imaged in PBS using the BioTek Cytation 5 Cell Imaging Multimode Reader (Agilent). The oxygen consumption rate values were normalized to the number of cells per well, calculated as imaged nuclei in each well.

### High-throughput amplicon sequencing of DNA samples

Sites of interest were amplified from isolated DNA samples and sequenced with the use of Illumina MiSeq system. The first round of PCR (PCR1) was performed with the use of primers amplifying the region of interest and containing partial Illumina adapters (Supplementary Table 3). Of total DNA, 200 ng was used for PCR1, performed with the use of Phusion Hot Start II High-Fidelity PCR Master Mix (Thermo Fisher Scientific) in 10 μl final volume using the following protocol: 98 °C for 30 s and then 16 cycles of 98 °C for 10 s, 56 °C for 30 s and 72 °C for 30 s, followed by a final 72 °C extension for 7 min. PCR1 was purified using AMPure XP beads (Beckman Coulter) according to the manufacturer’s protocol. PCR2, adding Illumina indexes, was performed using 2 µl of purified PCR1 product and amplified with Phusion Hot Start II High-Fidelity PCR Master Mix (Thermo Fisher Scientific) in a 10 μl final volume using the following protocol: 98 °C for 30 s and then 20 cycles of 98 °C for 10 s, 58 °C for 30 s and 72 °C for 30 s, followed by a final 72 °C extension for 7 min. PCR2 products were evaluated by electrophoresis in a 1.5% agarose gel, then up to 96 PCR2 products containing various Illumina barcode combinations were mixed and purified using the QIAquick PCR Purification Kit (Qiagen). The library concentration was measured using the Qubit dsDNA HS Assay Kit (Thermo Fisher Scientific) and further verified by qPCR using the NEBNext Library Quant Kit for Illumina (New England Biolabs). Libraries amplified with primers without a heterogeneity spacer were sequenced using an Illumina MiSeq system with 50-bp single-end reads at a final concentration of 12 pM with 5% PhiX spike-in (Illumina), or with 150-bp paired-end reads at a final concentration of 6 pM with 20% PhiX spike-in (Illumina). If primers with a heterogeneity spacer were used, libraries were sequenced at a concentration of 12 pM, without addition of the PhiX spike-in.

### High-throughput amplicon sequencing of RNA samples

Total RNA was extracted from cells using the RNeasy Mini Kit (Qiagen) and digested with ezDNase enzyme (Invitrogen). Of isolated RNA, 150 ng was used for reverse transcription, performed with SuperScript IV Reverse Transcriptase (Thermo Fisher Scientific) and target-specific reverse transcription primers listed in Supplementary Table 3. The obtained cDNA was used for preparing amplicon libraries as described above.

### Barnyard experiment to evaluate doublet rate

For evaluating the doublet rate, a SCI-LITE experiment was performed according to the detailed protocol (see Supplementary Protocol containing SCI-LITE detailed workflow.) with a minor modification: at the beginning of the experiment, HeLa and 293T cells were harvested and counted using the Vi-Cell XR Cell Viability Analyzer (Beckman Coulter). Equal numbers of HeLa and 293T cells were mixed and used for SCI-LITE. See Supplementary Discussion for more details on the analysis and interpretation of the Barnyard data.

### Initial processing and quality control of sequencing data

Illumina MiSeq runs were demultiplexed and converted to FASTQ format using bcl2fastq v2.20.0.422. The read quality in each FASTQ file was verified using the FASTQC tool v0.11.9 and aggregated into a single report per sequencing run using MultiQC v1.11.

### Analysis of high-throughput amplicon sequencing data of DNA and RNA samples

To analyse the bulk amplicon sequencing data, we used a custom script to iterate over each read in each FASTQ file and test for the presence of the expected alleles at the expected positions, keeping a running count of each allele in each sample. Counts from reads with sequences that did not match the expected edits or wild-type alleles were discarded. LHON heteroplasmy for each sample was calculated as the sum of all reads with LHON-containing alleles detected, divided by the sum of all non-discarded reads in that sample. The silent heteroplasmy was calculated as the sum of all reads that showed only the silent edit, and no LHON edit, divided by the sum of all non-discarded reads. The analyses were performed using Python v3.7.12, with the following modules: matplotlib v3.4.2, numpy v1.21.0, pandas v1.1.5, plotly v5.16.1, pysam v0.16.0.1, scikit-learn v0.23.1, scipy v1.7.0 and seaborn v0.11.1.

### Analysis of single-cell SCI-LITE data

We processed the SCI-LITE data using a custom Python pipeline that iterates over each read in each FASTQ file. The pipeline was run with Python v3.9.15, pysam v0.20.0, numpy v1.26.2, pandas v1.5.2, and matplotlib v3.8.2. The script parsed each read to extract the amplicon sequence, the reverse transcription barcode, the ligation barcode and the UMI sequence, before storing these in a table where information pertaining to each read composes one entry. Next, the script corrected all amplicon sequences, reverse transcription barcodes and ligation barcodes within a Hamming distance of 1 from an expected amplicon or barcode sequence, and filtered out any reads lacking matches to an expected amplicon sequence, reverse transcription barcode sequence or ligation barcode sequence. Cell IDs were constructed for the remaining reads by combining the Illumina barcode, the reverse transcription barcode and the ligation barcode, and then these reads were deduplicated for each cell ID based on the UMI sequences. On occasion, there were reads with the same UMI sequence but different *MT-ND4* alleles. To handle these cases, we filtered out any UMIs with fewer than three reads and then assigned each remaining UMI to an allele if at least 2/3 of the reads support that allele. Any UMIs with multiple alleles but without a 2/3 majority of their reads supporting one of those alleles were discarded. Once UMI alleles were resolved, valid cells were called by setting a UMI coverage threshold at the ‘knee’ of a plot of log_10_ UMI counts per cell versus the log_10_ ranking of cells by UMI count (Fig. 1c) besides in Fig. 1f where all cells with more than one UMI were analysed. Finally, heteroplasmy was calculated in each cell for each amplicon sequence type as the number of UMIs for each possible allele divided by the total number of UMIs belonging to that amplicon sequence type. As with the bulk sequencing data, for the LHON-edited and SILENT-edited experiments, UMIs showing both the missense and the silent edits on the same molecule were included in the calculation of the missense heteroplasmy and excluded from the silent heteroplasmy.

### Simple model of heteroplasmy dynamics

We created a simple simulation of a population of proliferating LHON-edited cells where heteroplasmy impacts cell fitness. In this simulation, we assumed that (1) the population doubles in size each day, (2) the heteroplasmy within a cell does not change, and (3) the relative probability that a cell will divide is a function of its heteroplasmy according to an inverse sigmoid function, which simulates a ‘biochemical threshold’ of heteroplasmy, beyond which cell fitness drops off rapidly (Extended Data Fig. 6d).

We initialized the day 0 distribution of heteroplasmy by sampling 2,500 cells, with replacement, from the empirically observed day 0 LHON-edited SCI-LITE data. To avoid having multiple cells with identical allele counts and heteroplasmy values if the same observed cell was sampled twice, we resampled the *MT-ND4* mtRNA molecules for each cell by making random draws from a multinomial distribution that was parameterized by the heteroplasmy fraction of the cell for LHON, SILENT and wild-type alleles. The number of multinomial draws for each cell was one of the fitted model parameters (described below) and was scaled for each cell by the number of observed UMIs in that cell divided by the maximum number of UMIs present in any observed day 0 cell. Thus, the number of simulated molecules is related to the amount of data supporting the heteroplasmy estimates in the observed cell. Once the multinomial draws were complete, then the LHON, SILENT and wild-type heteroplasmy values were recalculated based only on the simulated molecules.

Next, we simulated growth over a 15-day time course. At each simulated day, the culture doubles in size by drawing cells with replacement, and the probability of being selected is inversely related to its missense heteroplasmy according to an inverse sigmoid function (described below). Once the dividing cells were selected, the heteroplasmy was adjusted using the same multinomial sampling strategy that we used to generate the day 0 cells. To match the experimental protocol that generated the SCI-LITE time course data, cell culture passaging was simulated on days 3, 5, 8, 10 and 13 by sampling, without replacement, 5,000 cells before a 2-day interval between passages, or 2,500 cells before a 3-day interval between passages. Finally, a number of cells equal to the number of observed day 0 cells were sampled, with replacement, from the simulated culture at days 5, 10 and 15 to simulate the collection of SCI-LITE data. The fit of the model was assessed by comparing (1) the observed and simulated mean LHON and SILENT heteroplasmy estimates, and (2) the observed and simulated joint heteroplasmy distributions at each timepoint in the time series.

The model fitting procedure optimized four model parameters in parallel: three to define the inverse sigmoid function, and one to set the number of molecules to simulate per cell. We describe the inverse sigmoid function, which is defined by the following equation,

$$y(x)=\left(\left(1-d\right)\times \left[\frac{{10}^{-p\left(x-l\right)}}{1+{10}^{-p\left(x-l\right)}}\right]\right)+d$$

where *d* specifies the depth of the sigmoid below 1.0 (for example, function values range from *d* to 1.0; this largely defines the relative growth rate of a homoplasmic mutant cell compared with a homoplasmic wild-type cell), *l* specifies the location of the sigmoid inflection point on the *x* axis, and $p$ specifies the pitch of the transition from 1.0 to *d* (the combination of location and pitch defines the biochemical threshold, as well as the relative growth rate of a heteroplasmic mutant cell compared with a homoplasmic wild-type cell) (Extended Data Fig. 7a). We also tested different numbers of molecules to simulate per cell, a parameter that defines the precision of the heteroplasmy estimates in our cells. To estimate these four parameters, we took a random search approach, in which we created 20,000 random parameter sets, ran the models and chose the combination of parameters that led to modelling results most similar to our observed heteroplasmy distributions (Extended Data Fig. 7b). Our random search optimization minimized the combined mean squared error (MSE) between the simulated and observed missense and silent mean heteroplasmy values, and the simulated and observed joint heteroplasmy distributions, represented by 2D kernel density estimates, for days 5, 10 and 15. The two MSE calculations were combined by adding the 2D kernel density estimates MSE plus twice the mean heteroplasmy MSE. To smooth out noise in the random search, we set the combined MSE for each of the 20,000 iterations to be the average combined MSE of the 50 nearest neighbours in the search space. The model with the minimum smoothed combined MSE had an inverse sigmoid function with a depth (*d*) of 0.31, a pitch ($p$) of 4.95 and a location (*l*) of 0.75, and simulated 1,000 molecules per cell (Extended Data Fig. 7c,d).

Having identified optimal model parameters, we then ran the simulated growth experiment 100 times, each with a different random seed. We present the joint heteroplasmy distributions for one representative model (Extended Data Fig. 6b), and also plot the distributions of the 100 mean heteroplasmy values and compare them to the observed bulk heteroplasmy values (Extended Data Fig. 6c).

### Lineage tracing data analysis

The data from the lineage tracing SCI-LITE experiment were processed in a way similar to the other SCI-LITE experiments, with some modifications to account for the lineage tracing barcodes. During initial read processing, sequences were checked against the list of expected lineage barcodes with an error tolerance of 1 mismatch. Lineage barcode UMIs underwent the UMI allele assignment in the same way as described above, with a requirement for at least 3 reads supporting the UMI, and then assignment of the allele shown by at least 2/3 of the reads. When calling valid cells using the knee plot, we did not include lineage barcode UMIs in the UMI coverage per cell, because it is most critical to maximize coverage of the mtDNA alleles to acquire good heteroplasmy estimates, and including lineage barcode UMIs in the knee plot yielded some cells with high coverage of the lineage barcode but low coverage of the mtDNA alleles. After cells were called, and heteroplasmy per cell was calculated as described above, we additionally filtered for cells that had a lineage barcode. We found that it was most common for a cell to have UMIs showing a single lineage barcode; however, some cells showed multiple lineage barcodes, and we corrected these in a similar way to UMIs that showed multiple alleles. In the cells showing multiple lineage barcodes, it was often the case that one lineage barcode had an overwhelming fraction of the UMIs supporting it. We found that in such cases, the second most common barcode often had fewer than 10 UMIs supporting it. So, in each cell, we removed any lineage barcodes with fewer than 10 UMIs detected, and then additionally required that the number of UMIs supporting the most common lineage barcode in a cell be at least four times the number of UMIs supporting the second most common lineage barcode in that cell. Cells were assigned to the lineage barcodes passing these filtering steps, and otherwise were left unassigned to a lineage barcode.

### Kimura distribution fitting

First, we filtered our LHON and SILENT time course SCI-LITE data to remove cells with zero heteroplasmy, to focus on cells that had undergone editing. Next, we used the ‘heteroplasmy’ R package (v0.0.2.1; https://github.com/StochasticBiology/heteroplasmy-analysis)^37^ to fit our LHON and SILENT time course data to the two-parameter Kimura distribution, Kimura(*h*_*i*_|*p*,*b*), where *h*_*i*_ is the heteroplasmy at the current timepoint, *p* is the initial population heteroplasmy and *b* is the extent of drift. We fixed the initial population heteroplasmy, *p*, to be the mean heteroplasmy observed empirically at our day 0 timepoint, and then used maximum likelihood estimation to fit the drift parameter (*b*) based on the day 5 SCI-LITE heteroplasmy measurements. We fitted both our LHON-edited and SILENT-edited data, and compared our observed heteroplasmy distributions to the fitted Kimuras using the Kolmogorov–Smirnov test in the test_kimura_par() function, with num_MC = 100. The Kolmogorov–Smirnov *D* statistic highlights the poor fit of the Kimura to the LHON data (*D* = 0.05 versus *D* = 0.26 for SILENT edited versus LHON edited), and this difference remains if we instead fit the Kimura by minimizing the Kolmogorov–Smirnov distance (*D* = 0.05 versus *D* = 0.25 for SILENT versus LHON).

Note that using the day 0 data is critical to seeing the influence of selection; if we fit both *p* and *b* to day 5 data, then we find no difference in the Kimura fit between SILENT and LHON. In addition, fitting both parameters by minimizing the Kolmogorov–Smirnov distance yields fits with Kolmogorov–Smirnov *P* > 0.05 for both the SILENT and the LHON data.

For visualization, we used the dkimura() function from the kimura R package (v0.0.0.9001; https://github.com/lbozhilova/kimura) to compute the probability density function of our Kimura distributions, and numpy.histogram() with density=True to compute the empirical density distribution of our day 5 heteroplasmy estimates (Extended Data Fig. 5). The analysis was done with Python v3.10.12, matplotlib v3.8.0, numpy v1.24.4, pandas v2.1.1, rpy2 v3.5.11, seaborn v0.13.0, R v4.2.3, heteroplasmy v0.0.2.1 and kimura v0.0.0.9001.

### Reporting summary

Further information on research design is available in the Nature Portfolio Reporting Summary linked to this article.

## Online content

Any methods, additional references, Nature Portfolio reporting summaries, source data, extended data, supplementary information, acknowledgements, peer review information; details of author contributions and competing interests; and statements of data and code availability are available at 10.1038/s41586-024-07332-0.

### Supplementary information

Supplementary Information This file contains the Supplementary Discussion, Supplementary Figs 1–3, Supplementary Sequences, Supplementary Tables 2 and 3 and Supplementary References.

Reporting Summary

Supplementary Protocol SCI-LITE detailed protocol.

Supplementary Table 1 List of oligonucleotids used in SCI-LITE.

Supplementary Code SCI-LITE code supplement.

### Source data

Source Data Figs. 1–5 and Source Data Extended Data Figs. 2, 3, 5, 6

## Data Availability

The high-throughput sequencing data have been deposited in the NCBI Sequence Read Archive (PRJNA1046659, https://www.ncbi.nlm.nih.gov/bioproject/PRJNA1046659). Data from all other experiments are provided as a supplementary csv file. Source data are provided with this paper.

## Code availability

The SCI-LITE pipeline software is available in a public GitHub repository (https://github.com/MoothaLab/scilite-pipeline), and the version used to generate the results in the paper is provided as a supplementary file to this paper. The parameters and auxiliary data required to run the SCI-LITE pipeline on each of the experiments reported in the paper, along with the code to perform downstream analysis and make figures, are provided in a separate GitHub repository (https://github.com/MoothaLab/scilite-analysis).
