# Supplementary material for: Single-cell analysis reveals context-dependent, cell-level selection of mtDNA
Source: Nature. 2024 Apr 24;629(8011):458–66. doi: 10.1038/s41586-024-07332-0 (PMC11078733; doi:10.1038/s41586-024-07332-0)
Supplement: Supplementary file 1 — This file contains the Supplementary Discussion, Supplementary Figs 1–3, Supplementary Sequences, Supplementary Tables 2 and 3 and Supplementary References. [file 41586_2024_7332_MOESM1_ESM.pdf]

nature portfolio
https://doi.org/10.1038/s41586-024-07332-0

Supplementary information

Single-cell analysis reveals context-dependent, cell-level selection of mtDNA

In the format provided by the authors and unedited

Supplementary Information

Supplement to: Kotrys AV, Durham TJ, Guo XA, Vantaku VR, Parangi S, Mootha VK

Single-cell analysis reveals context-dependent, cell-level selection of mtDNA

Page number:

SUPPLEMENTARY DISCUSSION 2

HETEROTYPIC AND HOMOTYPIC DOUBLETS IN SCI-LITE 2

LIMITATIONS OF THE STUDY 3

SUPPLEMENTARY FIGURES 5

SUPPLEMENTARY FIGURE 1. 5

SUPPLEMENTARY FIGURE 2. 6

SUPPLEMENTARY FIGURE 3. 7

SUPPLEMENTARY SEQUENCES 8

AMINO ACID SEQUENCE OF THE RIGHT TALE OF SILENT DDCBE 8

AMINO ACID SEQUENCE OF THE LEFT TALE OF SILENT DDCBE 8

SUPPLEMENTARY TABLES 9

TABLE S1. SCI-LITE OLIGONUCLEOTIDES 9

TABLE S2. REAGENTS. 9

TABLE S3. PRIMERS USED FOR SANGER SEQUENCING, RT-QPCR AND AMPLICON LIBRARIES .. 10

SUPPLEMENTARY REFERENCES 11

Supplementary discussion

Heterotypic and homotypic doublets in SCI-LITE

During sequential split-pool rounds of SCI-LITE, it's possible that two cells will traverse the same path by random chance. This theoretical "collision rate," or "doublet rate," can be estimated based on the total number of barcode combinations and the number of cells in the experiment[22]. The estimated collision rate includes both heterotypic doublets (cells from two different cell lines that traveled together) and homotypic doublets (cells from the same cell line that traveled together). To experimentally assess doublet rates in our "barnyard experiment," in addition to mixing HeLa and 293T cells, we also analyzed each cell line separately, wherein each cell line was barcoded with predefined set of RT barcodes that we then used to unambiguously assign reads to either cell line. In such an experiment we should not observe heterotypic doublets consisting of mixed HeLa and 293T reads, as both cell lines are physically separated during the initial RT step. We found that the cells in these non-mixed arms of the experiment had no more than 29 UMIs per cell from the other cell type detected, and we set this as our threshold for calling heterotypic doublets in the mixed arm of the experiment. With this threshold, we identified 6 heterotypic doublets in our population of 748 mixed HeLa and 293T cells (a rate of ~0.8%). Based on our 2% theoretical collision rate, we would expect ~1.2% of reads to represent homotypic doublets. Homotypic doublets are of course harder to identify, because the colliding cells have the same allele. Many single-cell analysis pipelines assume that doublet cells should have higher UMI coverage than singlets because they have twice the genetic content, and thus filter out the cells with the highest UMI coverage as probable homotypic doublets. However, when we compared the coverage of our heterotypic doublets to the overall UMI count distribution, we found that they were generally not at the extreme end of the distribution. Hence, a simple UMI count filter would remove many true singlets. Since our expected homotypic doublet rate is so low, we chose not to implement a coverage-based filter to remove doublets in our analysis pipeline. In general, we cannot confidently identify doublets outside of the barnyard SCI-LITE experiment, because in our other experiments all cells are expected to have the

same alleles but at variable heteroplasmy levels. Reassuringly, the barnyard experiment shows that our doublet rate is low and consistent with our expected rate of about 2%.

Limitations of the study

We acknowledge several limitations of this study.

First, SCI-LITE is designed for targeted analysis of selected transcripts, and therefore it requires optimization for each new target (e.g., mtDNA heteroplasmy of interest, etc.) analogous to how qPCR is typically optimized for each target.

Second, while we find that SCI-LITE results in generating reliable data supported by bulk heteroplasmy estimates, we nonetheless observed a small degree of cross-contamination between analyzed samples that arise in a few specific ways. First, two cells can take the same path through the pooling-and-splitting steps and manifest as a doublet cell in the data analysis as described above. In all experiments we report here, we were able to minimize the impact of doublets on our experimental results in two ways 1) by using a sufficient diversity of barcodes to attain a theoretical doublet rate of no more than 2%, and 2) assigning specific RT barcode sequences to specific samples, which makes heterotypic doublets far less likely. A second form of cross-contamination is when the same UMI is associated with different mtDNA variants. We observed that such cross-contamination events are more probable when more PCR cycles are used to amplify the libraries. We therefore recommend keeping PCR cycles to minimum while amplifying SCI-LITE libraries. We also developed and applied a computational correction - described in detail in the Methods section - that can help correct this kind of cross-contamination and assign the correct allele to each UMI. A third, very minor source of cross-contamination (often just a single UMI per cell if it is present) is due to ambient RNA, which comes from UMIs generated by RNA molecules that leak into the solution, probably from damaged or dying cells[45], and that then pass through the same combination of wells in the pooling-and-splitting process as a valid cell (Supplementary Fig. 1).

Third, we have used DdCBE to model mtDNA heteroplasmy primarily in immortalized cell lines. While models of mtDNA disease have largely been lacking thus far, the advent of mtDNA base editing now makes it possible to generate primary cell models or animal models to further validate our results.

Fourth, our *in vivo* tumor xenograft model supports the importance of environment-dependent selection of mtDNA heteroplasmy that we observed in cultured cells, but deeper understanding of the mechanism requires further study. In our *in vivo* tumor model showing that the introduced complex I truncating mtDNA heteroplasmy confers a growth benefit to the tumor we used a thyroid follicular cell line (Nthy-ori 3-1) that has been immortalized with a single copy of the SV40 T antigen plasmid[33]. In recent analysis, missense variants in KMT2A, POLE and CHEK2 were identified in this cell line[34]. All of these variants have unspecified functional effects, but none of them are known to be drivers of tumorigenesis in Hürtle cell carcinomas[8,46]. Future studies are required to determine whether the introduced complex I mutation is required transiently or long-term in the tumor, and what advantage it confers to the cells for promoting tumor growth.

Supplementary figures

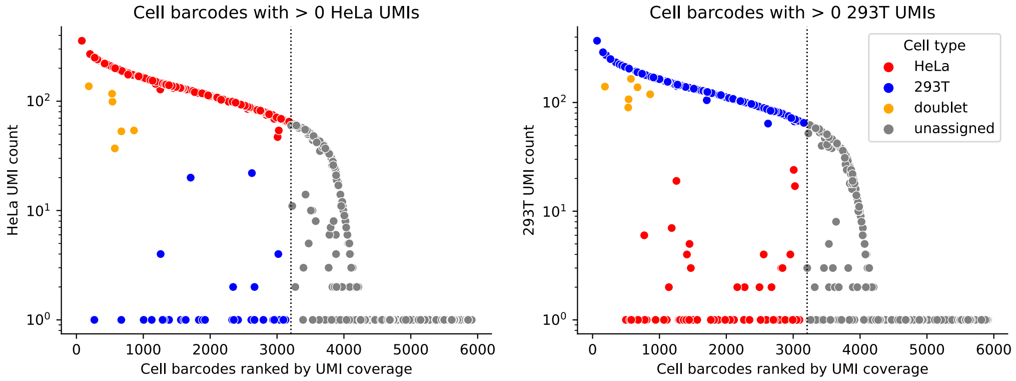

Supplementary Figure 1.
Low levels of ambient RNA contamination are present in SCI-LITE data, as shown in this analysis of the HeLa (**a**) and 293T (**b**) cells from the barnyard experiment (Fig.1 c-e). Ideally, HeLa cells would contain only HeLa alleles and 293T would contain only 292T alleles. We observed that some HeLa cells contain a low level of alternate 293T allele, and some 293T cells contain alternate HeLa allele. Most of the non-doublet cells with alternate alleles detected have just a single contaminating UMI, note that the y-axis is on a log scale.

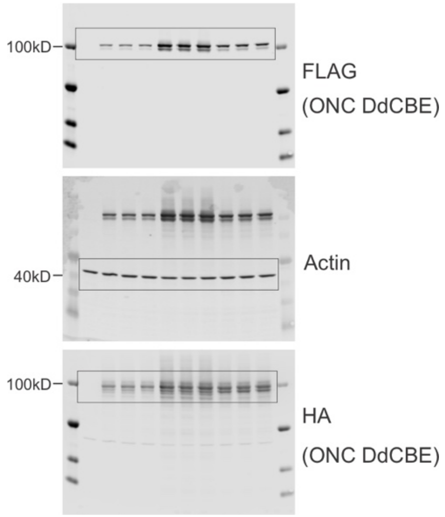

Supplementary Figure 2.
a, Uncropped western blot images. From Main Fig. 2b and Extended Data Fig. 2a.

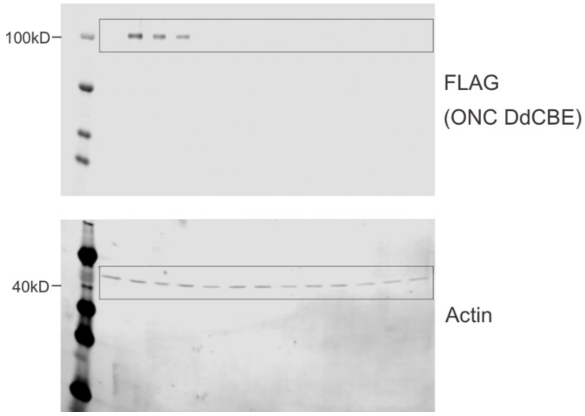

Supplementary Figure 3.
a, Uncropped western blot images from Extended Data Fig. 9c.

Supplementary sequences

Amino acid sequence of the right TALE of SILENT DdCBEs

MDIADLRTLGYSQQQQEKIKPKVRSTVAQHHEALVGHGFTHAHIVALSQHPAALGTVA
VKYQDMIAALPEATHEAIVGVGKQWSGARALEALLTVAGELRGPPLQLDTGQLLKIAKR
GGVTAVEAVHAWRNALTGAPLNLTPEQVVAIASNNGGKQALETVQALLPVLCQAHGLT
PQQVVAIASNNGGKQALETVQRLLPVLCQAHGLTPQQVVAIASNNGGKQALETVQRLL
PVLCQAHGLTPEQVVAIASHDGGKQALETVQALLPVLCQAHGLTPEQVVAIASNNGGK
QALETVQALLPVLCQAHGLTPEQVVAIASNIGGKQALETVQALLPVLCQAHGLTPEQVV
AIASNGGGKQALETVQRLLPVLCQAHGLTPEQVVAIASNGGGKQALETVQALLPVLCQ
AHGLTPQQVVAIASNIGGKQALETVQRLLPVLCQAHGLTPEQVVAIASNGGGKQALET
VQRLLPVLCQAHGLTPQQVVAIASNNGGKQALETVQRLLPVLCQAHGLTPQQVVAIAS
NIGGKQALETVQRLLPVLCQAHGLTPQQVVAIASNNGGRPALESIVAQLSRPDPALAAL
TNDHLVALACLGGRPALDAVKKGLG

Amino acid sequence of the left TALE of SILENT DdCBEs

MDIADLRTLGYSQQQQEKIKPKVRSTVAQHHEALVGHGFTHAHIVALSQHPAALGTVA
VKYQDMIAALPEATHEAIVGVGKQWSGARALEALLTVAGELRGPPLQLDTGQLLKIAKR
GGVTAVEAVHAWRNALTGAPLNLTPEQVVAIASNNGGKQALETVQALLPVLCQAHGLT
PQQVVAIASNIGGKQALETVQRLLPVLCQAHGLTPQQVVAIASNIGGKQALETVQRLLP
VLCQAHGLTPEQVVAIASNNGGKQALETVQALLPVLCQAHGLTPEQVVAIASHDGGKQ
ALETVQALLPVLCQAHGLTPEQVVAIASNGGGKQALETVQALLPVLCQAHGLTPEQVV
AIASNGGGKQALETVQRLLPVLCQAHGLTPEQVVAIASHDGGKQALETVQALLPVLCQ
AHGLTPQQVVAIASNIGGKQALETVQRLLPVLCQAHGLTPQQVVAIASHDGGRPALESI
VAQLSRPDPALAALTNDHLVALACLGGRPALDAVKKGLG

Supplementary tables

Table S1. SCI-LITE oligonucleotides. Table S1 is provided as a separate file.

Table S2. Reagents.

Reagent	Source	Identifier
**Antibodies**		
Mouse anti-FLAG (primary)	Sigma	F1804
Mouse anti-HA (primary)	Biolegend	901513
Rabbit anti-Actin (primary)	Cell Signaling	4970S
Goat anti-rabbit 680RD (secondary)	LI-COR	926–68071
Goat anti-mouse 800CW (secondary)	LI-COR	926-32210
**Chemicals**		
DMEM, high glucose, pyruvate	GIBCO	11995065
Sodium Pyruvate	GIBCO	11360070
Fetal Bovine Serum	GIBCO	26140079
Fetal Bovine Serum, dialyzed	GIBCO	26400044
DMEM, no glucose	GIBCO	11966025
D-(+)-Glucose	Sigma	G7021
D-(+)-Galactose	Sigma	G5388
Uridine	Sigma	U3750
Oligomycin A	Sigma	75351
Seahorse XF DMEM Medium pH 7.4	Agilent	103575-100
Glucose solution for Seahorse assay	Agilent	103577-100
L-glutamine	GIBCO	25030-081
Piericidin A	Cayman Chemical	15379
Antimycin A	Sigma	A8674
Carbonyl cyanide-4-(trifluoromethoxy)phenylhydrazone (FCCP)	Sigma	C2920
PBS	Life Technologies	10010049
UltraPure DNase/RNase-Free Distilled Water	Invitrogen	10977023
Blocking buffer	LI-COR	927-70050
GelGreen Nucleic Acid Stain	Sigma	SCT125
RIPA buffer	Boston BioProducts	BP-115
cOmplete Protease Inhibitor Cocktail	Roche	11697498001
Laemmli SDS-Sample Buffer	Boston BioProducts	BP-111R
Tris-glycine running buffer	BioRad	1610732
Bloting-Grade Blocker	BioRad	170-6404
Poly-L-lysine hydrobromide	Sigma	P6282
Hoechst 33342 Solution	Thermo Fisher Scientific	H3570
AMPure XP beads	Beckman Coulter	A63881
Bovine serum albumin	Sigma	A3294
Lipofectamine 2000 reagent	Invitrogen	11668
iQ SYBR Green Supermix	Bio-Rad	1708880
**Commercial assays**		
QIAquick PCR Purification Kit	Qiagen	28104
DNeasy Blood & Tissue Kit	Qiagen	69506

QIAquick Gel Extraction Kit	Qiagen	28704
RNeasy Mini Kit	Qiagen	74106
Phusion Hot Start II High-Fidelity PCR Master Mix	Thermo Fisher Scientific	F565L
Trans-Blot Turbo Midi 0.2 µm Nitrocellulose Transfer Packs	BioRad	1704159
Novex WedgeWell 4 to 20%, Tris-Glycine, 1.0 mm, 15 wells, Mini Protein Gels	Invitrogen	XP04205BOX
GeneRuler Low Range DNA Ladder	Thermo Fisher Scientific	SM1193
SuperScript IV Reverse Transcriptase	Invitrogen	18090010
ezDNase Enzyme	Invitrogen	11766051
Qubit dsDNA HS Assay Kit	Thermo Fisher Scientific	Q32851
NEBNext Library Quant Kit for Illumina	New England Biolabs	E7630S
MiSeq Reagent Kit v2, 50 cycles	Illumina	MS-102-2001
MiSeq Reagent Kit v3, 150 cycles	Illumina	MS-102-3001
PhiX Control v3 Library	Illumina	FC-110-3001
**Cell lines**		
293T	ATCC	CRL-3216
HeLa	ATCC	CCL-2
K562	ATCC	CRL-243
Nthy-ori 3-1	ECACC	90011609
**Recombinant DNA**		
DdCBE installing truncating mt-ND4 mutation, right side TALE	Addgene	#157843
DdCBE installing truncating mt-ND4 mutation, left side TALE	Addgene	#157844
DdCBE installing LHON-associated mt-ND4 mutation, right side TALE	Addgene	#179686
DdCBE installing LHON-associated mt-ND4 mutation, left side TALE	Addgene	#179682
DdCBE installing SILENT mt-ND4 mutation, right side TALE	Liu lab / Broad Institute	
DdCBE installing SILENT mt-ND4 mutation, left side TALE	Liu lab / Broad Institute	

Table S3. Primers used for Sanger sequencing, RT-qPCR and amplicon libraries.

Gene	Sequence		Application
	Forward primer	Reverse primer	
MT-ND4 (SNP)	GCAAACTCAAACTACGAACGC	AGGTGTATGAACATGAGG	PCR and Sanger
MT-ND4 (ONC)	TCGTCGGCAGCGTCAGATGTGTATAAGAGACAG GAACTCTCTGTGCTAGTAAC	GTCTCGTGGGCTCGGAGATGTGTATAAGAGACAG CGTAAGCCTCTGTTGTCAG	DNA amplicon libraries, PCR1

MT-ND4 (LHON/SILENT)	TCGTCGGCAGCGTCAGATGTGTATAAGAGACAG GCCATTCTCATCCAAACC	GTCTCGTGGGCTCGGAGATGTGTATAAGAGACAG GTATGTTGAGTCCTGTAAGTAG	DNA amplicon libraries, PCR1
MT-ND4 (LHON/SILENT)	TCGTCGGCAGCGTCAGATGTGTATAAGAGACAG GCCATTCTCATCCAAACC	GTCTCGTGGGCTCGGAGATGTGTATAAGAGACAG CAGACGTGTGCTCTTCCGA	RNA amplicon libraries, PCR1
MT-ND4	CAGACGTGTGCTCTTCCGANNNNNNNNNNgtatgttgagtcctgtaagt		Target-specific RT primer
MT-ND4	TCGTCGGCAGCGTCAGATGTGTATAAGAGACAG TCTCTGTGCTAGTAACCACG		RT-qPCR

Marked in red are Illumina partial adapter sequences.

Supplementary references

45. Janssen, P. et al. The effect of background noise and its removal on the analysis of single-cell expression data. Genome Biology 24, 140 (2023).

46. Gopal, R. K. et al. Effectors Enabling Adaptation to Mitochondrial Complex I Loss in Hürthle Cell Carcinoma. Cancer Discov 13, 1904–1921 (2023).
