# Supplementary material for: Single-cell analysis reveals context-dependent, cell-level selection of mtDNA
Source: Nature. 2024 Apr 24;629(8011):458–66. doi: 10.1038/s41586-024-07332-0 (PMC11078733; doi:10.1038/s41586-024-07332-0)
Supplement: Supplementary file 2 — Reporting Summary [file 41586_2024_7332_MOESM2_ESM.pdf]

nature portfolio

Corresponding author(s): Vamsi K. Mootha

Last updated by author(s): Feb 15, 2024

Reporting Summary

Nature Portfolio wishes to improve the reproducibility of the work that we publish. This form provides structure for consistency and transparency in reporting. For further information on Nature Portfolio policies, see our Editorial Policies and the Editorial Policy Checklist.

Statistics

For all statistical analyses, confirm that the following items are present in the figure legend, table legend, main text, or Methods section.

n/a	Confirmed	
☐	☒	The exact sample size (*n*) for each experimental group/condition, given as a discrete number and unit of measurement
☐	☒	A statement on whether measurements were taken from distinct samples or whether the same sample was measured repeatedly
☐	☒	The statistical test(s) used AND whether they are one- or two-sided<br>*Only common tests should be described solely by name; describe more complex techniques in the Methods section.*
☒	☐	A description of all covariates tested
☒	☐	A description of any assumptions or corrections, such as tests of normality and adjustment for multiple comparisons
☐	☒	A full description of the statistical parameters including central tendency (e.g. means) or other basic estimates (e.g. regression coefficient) AND variation (e.g. standard deviation) or associated estimates of uncertainty (e.g. confidence intervals)
☐	☒	For null hypothesis testing, the test statistic (e.g. *F*, *t*, *r*) with confidence intervals, effect sizes, degrees of freedom and *P* value noted<br>*Give P values as exact values whenever suitable.*
☒	☐	For Bayesian analysis, information on the choice of priors and Markov chain Monte Carlo settings
☒	☐	For hierarchical and complex designs, identification of the appropriate level for tests and full reporting of outcomes
☒	☐	Estimates of effect sizes (e.g. Cohen's *d*, Pearson's *r*), indicating how they were calculated

Our web collection on statistics for biologists contains articles on many of the points above.

Software and code

Policy information about availability of computer code

Data collection: High-throughput sequencing data was collected using Illumina MiSeq and NextSeq instruments.

Data analysis: High-throughput sequencing runs were demultiplexed and converted to FASTQ format using bcl2fastq v2.20.0.422. The read quality in each FASTQ file was verified using the FASTQC tool v0.11.9 and aggregated into a single report per sequencing run using MultiQC v1.11. We used custom scripts to analyze bulk amplicon sequencing data as well as single-cell SCI-LITE data. The SCI-LITE pipeline was run with Python 3.9.15, pysam 0.20.0, numpy 1.26.2, pandas 1.5.2, and matplotlib 3.8.2. Most of the analyses was done using Python v3.7.12, with the following modules: matplotlib 3.4.2, numpy 1.21.0, pandas 1.1.5, plotly 5.16.1, pysam 0.16.0.1, scikit-learn 0.23.1, scipy 1.7.0, seaborn 0.11.1. The exception was the Kimura analysis, which was done with Python 3.10.12, matplotlib 3.8.0, numpy 1.24.4, pandas 2.1.1, rpy2 3.5.11, seaborn 0.13.0, R 4.2.3, heteroplasmy 0.0.2.1, and kimura 0.0.0.9001.

The SCI-LITE pipeline software is available in a public GitHub repository (https://github.com/MoothaLab/scilite-pipeline), and the version used to generate the results in the manuscript is provided as a supplementary file to this manuscript. The parameters and auxiliary data required to run the SCI-LITE pipeline on each of the experiments reported in the paper, along with the code to do downstream analysis and make figures, is provided in a separate GitHub repository (https://github.com/MoothaLab/scilite-analysis).

SH800S software version 2.1.6 and FCS Express software version 7.10.0007 were used to analyze FACS data.

For manuscripts utilizing custom algorithms or software that are central to the research but not yet described in published literature, software must be made available to editors and reviewers. We strongly encourage code deposition in a community repository (e.g. GitHub). See the Nature Portfolio guidelines for submitting code & software for further information.

Data

Policy information about availability of data

All manuscripts must include a data availability statement. This statement should provide the following information, where applicable:

- Accession codes, unique identifiers, or web links for publicly available datasets
- A description of any restrictions on data availability
- For clinical datasets or third party data, please ensure that the statement adheres to our policy

High-throughput sequencing data are deposited in the NCBI Sequence Read Archive SRA project (PRJNA1046659)

Human research participants

Policy information about studies involving human research participants and Sex and Gender in Research.

Reporting on sex and gender	N/A
Population characteristics	N/A
Recruitment	N/A
Ethics oversight	N/A

Note that full information on the approval of the study protocol must also be provided in the manuscript.

Field-specific reporting

Please select the one below that is the best fit for your research. If you are not sure, read the appropriate sections before making your selection.

☒ Life sciences ☐ Behavioural & social sciences ☐ Ecological, evolutionary & environmental sciences

For a reference copy of the document with all sections, see nature.com/documents/nr-reporting-summary-flat.pdf

Life sciences study design

All studies must disclose on these points even when the disclosure is negative.

Sample size	No statistical methods were used to predetermine sample size. All experiments involving cell samples were evaluated in at least three independent biological replicates (n>=3) except in Supplementary Fig. 2e where in the low heteroplasmic cells at day 5 n=2. We and others have found these sample sizes to be sufficient to yield reproducible results.
Data exclusions	No data was excluded.
Replication	Independent biological triplicates were analyzed. All experiments were repeated at least once and all attempts were successful.
Randomization	The experiments were not randomized. In every experiment all biological replicates were treated identically therefore randomization was not necessary.
Blinding	The investigators were not blinded during experiments and outcome assessment. In every experiment all biological replicates were treated identically therefore blinding was not necessary.

Reporting for specific materials, systems and methods

We require information from authors about some types of materials, experimental systems and methods used in many studies. Here, indicate whether each material, system or method listed is relevant to your study. If you are not sure if a list item applies to your research, read the appropriate section before selecting a response.

Materials & experimental systems

n/a	Involved in the study
☐	☒ Antibodies
☐	☒ Eukaryotic cell lines
☒	☐ Palaeontology and archaeology
☐	☒ Animals and other organisms
☒	☐ Clinical data
☒	☐ Dual use research of concern

Methods

n/a	Involved in the study
☒	☐ ChIP-seq
☐	☒ Flow cytometry
☒	☐ MRI-based neuroimaging

Antibodies

Antibodies used	Mouse anti-FLAG (Sigma, F1804, 1:1,000 dilution), mouse anti-HA (Biolegend, 901513, 1:6,000 dilution), rabbit anti-Actin (Cell Signaling, 4970S, 1:1,000 dilution), Goat anti-rabbit 680RD (Li-Cor, 926–68071, 1:10,000), Goat anti-mouse 800CW (Li-Cor, 926-32210, 1:10,000)
Validation	Mouse anti-FLAG: validated by manufacturer by western blot against crude cell lysates from E. coli and mammalian cells<br>Mouse anti-HA: validated by manufacturer by western blot against cell lysates from CHO and CHO HA stable cells<br>Rabbit anti-Actin: validated by manufacturer by western blot against cell lysates from HeLa, NIH/3T3, C6, COS-7, BAEC and PAEC cells as well as recombinant Actin isoforms<br>Goat anti-rabbit 680RD and Goat anti-mouse 800CW, IRDye-labelled secondary antibodies have been tested and qualified for Western blot assay application by manufacturer

Eukaryotic cell lines

Policy information about cell lines and Sex and Gender in Research

Cell line source(s)	ATCC 293T (CRL-3216), ATCC HeLa (CCL-2), ATCC K562 (CRL-243), ECACC Nthy-ori 3-1 (90011609)
Authentication	Cells were authenticated by STR profiling by the supplier.
Mycoplasma contamination	All cell lines were tested negative for mycoplasma.
Commonly misidentified lines (See ICLAC register)	No commonly misidentified cell lines were used.

Animals and other research organisms

Policy information about studies involving animals; ARRIVE guidelines recommended for reporting animal research, and Sex and Gender in Research

Laboratory animals	Female NSG (NOD.Cg-Prkdcscid Il2rgtm1Wjl/SzJ) mice of 4-6 weeks of age purchased from The Jackson Laboratory (RRID: IMSR_JAX:005557)
Wild animals	No wild animals were used in the study.
Reporting on sex	Results do not apply to one sex only. Female mice were used in the study as thyroid cancers are more predominant in women than men.
Field-collected samples	No field-collected samples were used in the study.
Ethics oversight	Massachusetts General Hospital Institutional Animal Care and Use Committee

Note that full information on the approval of the study protocol must also be provided in the manuscript.

Flow Cytometry

Plots

Confirm that:

☒ The axis labels state the marker and fluorochrome used (e.g. CD4-FITC).

☒ The axis scales are clearly visible. Include numbers along axes only for bottom left plot of group (a 'group' is an analysis of identical markers).

☒ All plots are contour plots with outliers or pseudocolor plots.

☒ A numerical value for number of cells or percentage (with statistics) is provided.

Methodology

Sample preparation	293T cells transfected with DdCBEs harboring fluorescent reporters were harvested 24h after lipofection and used for FACS. Sorting was performed based on the intensity of eGFP/mCherry fluorescence. 293T cells stained with the CellTrace reagent were harvested 4 days after staining and sorted based on the CellTrace intensity (AF647 channel). Before sort, cells were resuspended in PBS and filtered to remove debris.
Instrument	Sony SH800S Cell Sorter was used.
Software	SH800S software version 2.1.6 and FCS Express software version 7.10.0007 were used.
Cell population abundance	Sort settings with at least 95% purity were used. The abundance of low, medium and high edited cells was 33.01%, 14.58% and 25.36%, respectively.
Gating strategy	Negative control to establish GFP-/mCherry- gates was achieved using cells that were not transfected with GFP and mCherry plasmids. Positive control to establish GFP+/mCherry+ gates was achieved using cells that were transfected with GFP and mCherry plasmids. Established gates were used for all subsequent samples, so that all samples were gated in the exactly same way.

☒ Tick this box to confirm that a figure exemplifying the gating strategy is provided in the Supplementary Information.
