# Supplementary material for: Single-cell analysis reveals context-dependent, cell-level selection of mtDNA
Source: Nature. 2024 Apr 24;629(8011):458–66. doi: 10.1038/s41586-024-07332-0 (PMC11078733; doi:10.1038/s41586-024-07332-0)
Supplement: Supplementary file 3 — SCI-LITE detailed protocol. [file 41586_2024_7332_MOESM3_ESM.pdf]

SCI-LITE detailed workflow

Supplement to: Kotrys AV, Durham TJ, Guo XA, Vantaku VR, Parangi S, Mootha VK

Single-cell analysis reveals context-dependent, cell-level selection of mtDNA

SCI-LITE (Single-cell Combinatorial Indexing Leveraged to Interrogate Targeted Expression) is a high throughput sequencing technique that enables profiling selected transcripts in millions of single cells. We harnessed combinatorial indexing (Rosenberg et al., Science, 2018, Cao et al., Nature, 2019) to develop a high-throughput, scalable and cost-effective tool for investigating mitochondrial DNA (mtDNA) heteroplasmy in single cells.

Workflow overview

1. Collecting and fixing cells
2. Permeabilization
3. Indexed reverse transcription
4. Indexed ligation
5. Lysis
6. Nested PCR
7. Indexed PCR
8. Library purification

Buffers

- Fixing buffer: 4% v/v formaldehyde in PBS (make fresh)
- Quenching buffer: 1M Tris-HCl pH 8.0
- Permeabilization buffer: 100 mM Tris-HCl pH 7.4, 150 mM NaCl, 3 mM $MgCl_2$, 0.2 mg ml^{-1} BSA, 0.1% v/v NP-40
- Cell buffer (CB): 100 mM Tris-HCl pH 7.4, 100 mM NaCl, 3 mM $MgCl_2$
- Cell buffer with BSA (CBB): 100 mM Tris-HCl pH 7.4, 100 mM NaCl, 3 mM $MgCl_2$, 0.2 mg ml^{-1} BSA

Filter all buffers and store at 4°C. Before use add SUPERaseIn RNase inhibitor (final 200 U/ml), store on ice.

Preparation of indexing plates

- RT plates: To each well of a 96-well plate add 2 µl of 10 mM dNTP and 2 µl of 100 µM barcoded RT primers. Plates can be prepared in advance and stored at -20°C.
- Ligation plates: To each well of a 96-well plate add 1.1 µl of 100 µM linker strand, 1.2 µl of 100 µM barcoded ligation oligo and 7.7 µl of oligo buffer (50 mM Tris-HCl pH 7.6, 50 mM NaCl). Seal plates and heat to 95°C for 2 min and cool down to 20°C at a rate of -0.1°C s^{-1}. Plates can be prepared in advance and stored at 4°C.
- Blocking strands: Prepare 26.4 µM blocking strands in 1 X T4 ligase buffer. Prepare 10 µL of blocking strand solution per well.

Collision rate calculation

We use collision rate estimation from Ma et al., Cell, 2020. The expected number of collisions for N cells with the total number of barcode combination D is: N-D+D((D-1)/D)^N. For example, using 96 indexes per each barcoding rounds results in 96 x 96 x 96 = 884, 736 combinations. With 40,000 cells used for sequencing, the number of collisions is 40000 - 884736 + 884736 x ((884736 - 1) / 884736) ^ 40000 = 890,73. The estimated collision rate is 890,73 / 40000 = 2.23%.

Detailed protocol

1. Collecting and fixing cells

   a) If working with adherent cells aspirate media, wash cells with PBS and trypsinize.
   b) Move detached cells to 50 ml conical Falcon tubes and centrifuge 400 x g, 3 min at 4°C.
   c) Remove supernatant and wash cells with PBS, centrifuge 400 x g, 3 min at 4°C.
   d) Resuspend cells in 1 ml PBS and transfer to 2 ml round bottom LoBind tubes.
   e) Centrifuge 400 x g, 3 min at 4°C and remove supernatant.
   f) Add 0.25 ml fixing buffer, mix by pipetting, and incubate on ice for 10 min.
   g) Add quenching buffer to a final concentration of 125 mM, mix by pipetting and incubate on ice for 5 min.
   h) Centrifuge 500 x g, 3 min at 4°C.

2. Permeabilization

   a) Remove supernatant and add 0.25 ml permeabilization buffer, mix by pipetting and incubate on ice for 3 min.
   b) Centrifuge 500 x g, 3 min at 4°C, remove supernatant and wash with 0.25 ml CBB.
   c) Centrifuge 600 x g, 2 min at 4°C, remove supernatant and resuspend cells in 2.25 ml CB.

3. Indexed reverse transcription

   a) Remove prepared RT plates from the freezer and let thaw completely.
   b) Add 22 μl cell suspension per well, mix 5 times up and down.
   c) Incubate the plate at 55 °C for 5 min. Immediately place on ice.
   d) Add 14 μl RT mix per well, mix 5 times up and down and start RT reaction.
   e) Add 60 μl CBB to each well and pool cells from all wells into the 5 ml Lo-bind eppendorf tube or sterile reagent reservoir.
   f) Centrifuge cells 600 x g, 2 min at 4°C, remove supernatant and wash cells with 0.5 ml CBB.
   g) Use cells directly for ligation or store at 4°C (STOPPING POINT).

RT MIX	(μl)
5 X Superscript IV First-Strand Buffer	8.0
DTT	2.0
SuperScript IV reverse transcriptase	1.5
RNaseOUT Recombinant Ribonuclease Inhibitor	1.5
H_2O	1.0
total vol	14

RT Reaction	
Time	Temp

02:00	4
02:00	10
02:00	20
02:00	30
02:00	40
05:00	50
07:00	55
hold	12

4. Indexed ligation

a) Remove supernatant and resuspend cells in 1.9 ml of 1 X NEBuffer 3.1.
b) Add 1.9 ml of ligation mix and mix by pipetting, use 5 ml Lo-bind eppendorf tube or sterile reagent reservoir.
c) Add 40 μl cell suspension per well of prepared ligation plate, mix 5 times up and down.
d) Incubate the plate at 37 °C for 30 min.
e) Add 10 μl blocking strands per well, mix 5 times up and down and incubate the plate at 37 °C for 30 min.
f) Add 40 μl CBB to each well and pool cells from all wells into the 5 ml Lo-bind eppendorf tube or sterile reagent reservoir.
g) Centrifuge cells 600 x g, 2 min at 4°C, remove supernatant and wash cells twice with 0.5 ml CBB, repeat centrifugation step.
h) Resuspend cells in 0.5 ml CBB and use 10 μl of cell suspension for counting with the use of Neubauer cell counting chamber.
i) Dilute cell suspension in CBB buffer to desired concentration chosen based on the calculated collision rate (when using 96x96x96 barcodes resuspend cells to the final concentration of 104,167 cells ml^{-1})

Ligation MIX	(μl)
10 X T4 ligase buffer	5.0
T4 DNA ligase (120U/uL)	3.0
H2O	14.0
total vol	22.0

5. Lysis

a) Add 10 μl of DNA extraction solution per well of 96 wells plate.
b) Add 4 μl of cell suspension per well containing DNA extract solution and mix by pipetting.
c) Incubate the plate at 65°C for 45 min followed by incubation at 98°C for 5 min.
d) Use lysates directly for PCR or store at 4°C (STOPPING POINT).

6. Nested PCR

a) Prepare nested PCR mix and add 8 μl per well of 96 wells plate.
b) Add 2 μl of lysate per well, seal plate, vortex briefly and spin plate 900 x g, 2 min at RT.
c) Start nested PCR reaction.
e) After the reaction is finished, perform purification of PCR products using AMPure XP beads. Use 1.1 X beads per 1 X reaction volume. Follow manufacturer's protocol. Perform elution in 12 μl of sterile water (STOPPING POINT).

Nested PCR MIX	(µl)
Phusion Hot Start II High-Fidelity PCR Master Mix	5.00
Forward primer (10 µM)	0.25
Reverse primer (10 µM)	0.25
H2O	2.50
total vol	10.00

PCR thermal profile:

Program	Time	Temp	Cycles
Pre-incubation			1
	00:30	98	
Amplification			(determine number of cycles by qPCR)
	00:10	98	
	00:30	56	
	00:30	72	
Final extension			1
	07:00	72	
Cooling			1
		4	

7. Indexed PCR

a) Prepare indexed PCR mix and add 8 µl per well of 96 wells plate.
b) Add 2 µl of purified nested PCR, seal plate, vortex briefly and spin plate 900 x g, 2 min at RT.
c) Start indexed PCR reaction.

Indexed PCR MIX	(µl)
Phusion Hot Start II High-Fidelity PCR Master Mix	5.00
Illumina i5 Index	0.25
Illumina i7 Index	0.25
H2O	2.50
total vol	10.00

PCR thermal profile:

Program	Time	Temp	Cycles
Pre-incubation			1
	00:30	98	
Amplification			(determine number of cycles by qPCR)
	00:10	98	
	00:30	58	
	00:30	72	
Final extension			1

	07:00	72	
Cooling			1
		4	

8. Library purification

   a) Pool PCR products from all wells and resolve in 1.5% agarose gel in TBE with GelGreen.
   b) Cut out the bands from the gel corresponding to the library size and purify using QIAquick Gel Extraction Kit. Elute product in 60 μl of sterile water (STOPPING POINT).
   c) Measure library concentration: use Qubit for estimated library concentration, perform qPCR using NEBNext Library Quant Kit for Illumina for detailed library concentration assessment (STOPPING POINT).
   d) Dilute libraries and sequence using the Illumina MiSeq or NextSeq system. Read configuration depends on the analyzed variants. Exemplary read configuration for ONC heteroplasmy: Read 1: 50 cycles, Read 2: 100 cycles, Index reads: 8 cycles. If not using primers with heterogeneity spacer we recommend sequencing SCI-LITE libraries at the 8 pM concentration with addition of 20% PhiX spike-in. If primers with heterogeneity spacer are used, we recommend sequencing SCI-LITE libraries at the 12 pM concentration, without addition of PhiX spike-in.

Equipment and consumables:

- Ice buckets
- Freezer and refrigerator
- Refrigerated microcentrifuge that holds 1.5 ml tubes (for example Eppendorf, 5424R)
- Refrigerated multifuge that holds 50 ml tubes and 96-well plates (for example Beckman Coulter, Allegra X-15R)
- PCR machine (for example Bio-Rad, C1000 Touch Thermal Cycler)
- qPCR machine (for example Bio-Rad, CFX Opus 384 Real-Time PCR System)
- MiSeq / NextSeq platform (Illumina)
- Chemical fume hood
- Single and multi-channel pipettes
- Wide orifice pipette tips
- 50 ml Falcon tubes
- 2 ml and 5 ml LoBind conical tubes (VWR International, 80077-234)
- 96-well LoBind plates (VWR International, 76204-386)
- Sterile reagent reservoirs (VWR International, 89094-662)
- Clear adhesive seals (Applied Biosystems, 4306311)
- Neubauer cell counting chamber (for example Sigma BR717820)

Ragents and chemicals:

Reagent	Source	Identifier
SuperScript IV Reverse Transcriptase	Life Technologies	18090050
T4 DNA ligase	New England Biolabs	M0202L
RNaseOUT Recombinant Ribonuclease Inhibitor	Invitrogen	10777019
SUPERase•In RNase Inhibitor	Invitrogen	AM2696
16% Formaldehyde (w/v), Methanol-free	Pierce	28906
dNTP mix	New England Biolabs	N0447L

BSA, Molecular Biology Grade	New England Biolabs	B9000S
NEBuffer 3.1	New England Biolabs	B7203S
1M Tris-HCl pH 8.0	Invitrogen	15568025
5M NaCl RNase-free	Invitrogen	AM9760G
1M $MgCl_2$	Sigma	M1028
NP-40 Surfact-Amps Detergent Solution	Thermo Scientific	28324
GeneRuler Low Range DNA Ladder, ready-to-use	Thermo Scientific	SM1193
Phusion Hot Start II High-Fidelity PCR master mix	Thermo Scientific	F565L
AMPure XP beads	Beckman Coulter	A63881
QuickExtract DNA Extraction Solution	Lucigen	QE09050
PBS	Life Technologies	10010049
UltraPure DNase/RNase-Free Distilled Water	Invitrogen	10977023
GelGreen Nucleic Acid Stain	Sigma	SCT125
QIAquick Gel Extraction Kit	Qiagen	28704
Qubit dsDNA HS Assay Kit	Thermo Scientific	Q32851
NEBNext Library Quant Kit for Illumina	New England Biolabs	E7630S
MiSeq Reagent Kit v3, 150 cycles	Illumina	MS-102-3001
PhiX Control v3 Library	Illumina	FC-110-3001
Nextera XT Index Kit v2 Set A	Illumina	FC-131-2001

Estimated costs of major enzymes and reagents:

**Reagent**	**Cost per μL ($)**	**Cost per plate ($)**
SuperScript IV Reverse Transcriptase	8.540	1229.76
T4 DNA ligase	1.040	299.52
RNaseOUT Recombinant Ribonuclease Inhibitor	1.664	239.62
SUPERase In RNase Inhibitor	0.970	29.10
dNTP	0.065	12.53
Phusion Hot Start II High-Fidelity PCR master mix	0.035	34.02
AMPure XP beads	0.023	23.91
QuickExtract DNA Extraction Solution	0.007	7.01
**Total cost per plate ($)**		1875.46
**Total cost per cell** (when using 384 barcodes) **($)**		0.003
