# Supplementary figures and images for: Single-cell analysis reveals context-dependent, cell-level selection of mtDNA
Source: Nature. 2024 Apr 24;629(8011):458–66. doi: 10.1038/s41586-024-07332-0 (PMC11078733; doi:10.1038/s41586-024-07332-0)

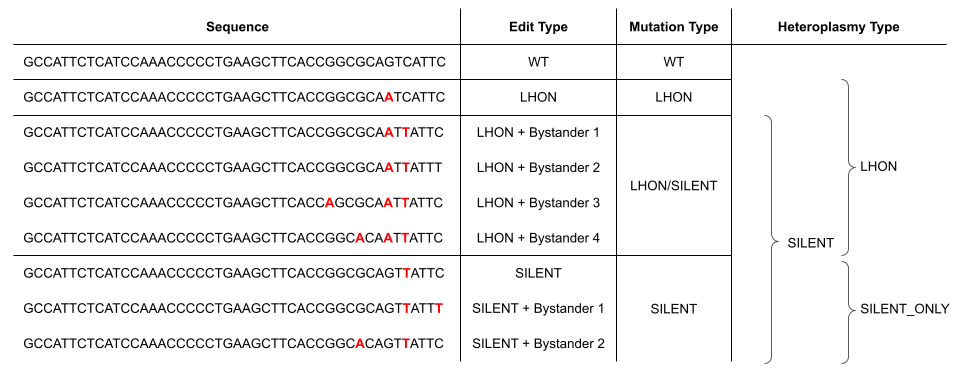

Supplement: Supplementary file 5 — SCI-LITE code supplement. [file 41586_2024_7332_MOESM5_ESM.zip › scilite_code_supplement/scilite-pipeline/fig_edit_mut_het_mapping.png]
